# “Cation-Stitching Cascade”: exquisite control of terpene cyclization in cyclooctatin biosynthesis

**DOI:** 10.1038/srep18471

**Published:** 2015-12-18

**Authors:** Hajime Sato, Kazuya Teramoto, Yui Masumoto, Noriyuki Tezuka, Kenta Sakai, Shota Ueda, Yusuke Totsuka, Tetsuro Shinada, Makoto Nishiyama, Chao Wang, Tomohisa Kuzuyama, Masanobu Uchiyama

**Affiliations:** 1 Graduate School of Pharmaceutical Sciences, The University of Tokyo, 7-3-1 Hongo, Bunkyo-ku, Tokyo 113-0033, Japan; 2 Elements Chemistry Laboratory, RIKEN, and RIKEN Center for Sustainable Resource Science (Wako campus), 2-1 Hirosawa, Wako-shi, Saitama-ken 351-0198, Japan; 3 Biotechnology Research Center, The University of Tokyo, 1-1-1 Yayoi, Bunkyo-ku, Tokyo 113-8657, Japan; 4 Department of Material Science, Graduate School of Science, Osaka City University, Sugimoto, Sumiyoshi-ku, Osaka 558-8585, Japan

## Abstract

Terpene cyclization is orchestrated by terpene cyclases, which are involved in the biosynthesis of various cyclic natural products, but understanding the origin and mechanism of the selectivity of terpene cyclization is challenging. In this work, we describe an in-depth mechanistic study on cyclooctatin biosynthesis by means of theoretical calculations combined with experimental methods. We show that the main framework of cyclooctatin is formed through domino-type carbocation transportation along the terpene chain, which we call a “cation-stitching cascade”, including multiple hydrogen-shifts and a ring rearrangement that elegantly determine the stereoselectivity.

Terpenoids or isoprenoids are a large and highly diverse group of natural products; over 55,000 terpenoids with a wide range of chemical structures (including monocyclic or multi-cyclic fused ring structures) and various biological activities have been reported to date^1^. This structural diversity is generated in the course of the cyclization reactions catalyzed by terpene cyclases, which provide remarkable examples of exquisite enzymatic control of the course of a chemical reaction cascade^2^,^3^,^4^.

We have established that CotB2, a diterpene cyclase^5^ identified in a soil-living filamentous bacterium *Streptomyces melanosprofaciens*, provides an especially elegant example of synthetic virtuosity and stereochemical control; CotB2 synthesizes the characteristic 5-8-5 fused ring system of cyclooctat-9-en-7-ol (**PD**), containing six chiral carbons, by cyclizing the achiral and acyclic C_20_ substrate (**SM**), geranylgeranyldiphosphate (GGDP)^5^. Among various diterpene cyclases, CotB2 specifically produces PD as a single product with the correct C–C bond connectivity and stereochemistry. Thus, CotB2 appears to precisely pre-organize the conformation of SM, control the conformations of all reactive intermediates, and properly regulate the reactivities of the cation intermediates in its active site pocket.

We previously proposed the mechanism of the CotB2-catalyzed reaction cascade (Fig. 1), based on a powerful combination of *in vivo* tracer experiments using uniformly ^13^C-labeled glucose and *in vitro* reactions of regiospecifically ^2^H-substituted **SM**^6^. The reaction cascade is divided into three parts, ring skeleton construction (Part A), hydrogen migrations (Part B), and carbon–carbon backbone rearrangement (Part C). Part A begins with CotB2-catalyzed dissociation of the pyrophosphate group followed by initial cyclization to produce a cation intermediate with a bicyclic 5–11 fused-ring skeleton. Subsequent 1,5-hydrogen-transfer from C8 to C15 provides an allylic cation, which undergoes intramolecular cyclization reaction with the C2-C3 olefin to form a cationic intermediate bearing the tricyclic 5-8-5 fused-ring system. The second part (Part B, Fig. 1) involves long-range cation transfer via multiple hydrogen shifts. The cationic intermediate likely undergoes a 1,3-H shift from C6 to C3 and a successive 1,5-H shift from C10 to C6 to generate the homoallylic cation. In Part C, formation of the cyclopropylcarbinyl cation, which features a C8–C9–C10 cyclopropyl ring, is followed by interconversion of cyclopropylcarbinyl cations. Finally, C7–C8 ring opening with capture of water by the homoallylic cation results in the formation of **PD**. Although our previous isotope tracing experiments and NMR spectroscopic investigations demonstrated the presence of the unusual carbon–carbon bond rearrangement, less is known about other intermediates and the mechanisms of other reactions on the pathway to the final product. For instance, in Part B, an alternative cascade involving two successive non-concerted 1,2-H shifts, firstly C2 to C3 yielding a cation at C2, then C6 to C2, could not be excluded in the previous work. Recent advances in theoretical methods, coupled with the enormous increase in available computational power, have enabled chemists to calculate transition structures and reaction pathways for complex molecules^7^,^8^,^9^,^10^. Density functional theory (DFT) combined with the AFIR (artificial force induced reaction) method and Reaction Plus method was employed herein to corroborate the experimental results and to comprehensively unveil this biosynthetic pathway.

All calculations were carried out at the M062X/6-31G** level^11^ using Gaussian 09^12^, GRRM11^13^, and Reaction Plus^14^ programs. The full reaction pathway for the conversion of geranylgeranyl diphosphate (GGDP: **SM**) to cyclooctat-9-en-7-ol (**PD**) and the energy diagram are presented in Figs 2 and 3, respectively. We will first discuss the CotB2-mediated carbocation formation and successive tricyclic skeleton construction part (Part A). Dissociation of the pyrophosphate of **SM** yields an allylic carbocation (**IM1**) partially stabilized by a cation-π interaction with a distal C10–C11 double bond^15^. Cation-mediated annulation proceeds smoothly to form a bicyclic intermediate (**IM2**), which undergoes sequential conformational change^16^ to give a more stable intermediate (**IM3**) with an activation energy of 12.2 kcal/mol. **IM3** contains two non-equivalent hydrogens at the C8 carbon atom. The α-hydrogen (downward) atom of the two hydrogens can migrate smoothly^17^ along the intrinsic reaction coordinate to the C15 carbocation with 6.6 kcal/mol stabilization energy for formation of the more stable allylic cation (**IM4**). **TS_3-4** is conformationally more favorable than **TS_3-4β** (1,5-β–H shift) by ca. 40 kcal/mol. The second annulation then occurs smoothly with an activation barrier of 5.5 kcal/mol to form the 5-8-5 fused-ring skeleton (**IM5**) with a large stabilization energy (ΔG = 13.3 kcal/mol). The overall gain to construct the tricyclic structure **IM5** is rather large (–25.5 kcal/mol from **IM1**). In Part B, multiple hydrogen shifts occur successively (**IM5→IM6→IM7→IM8→IM9**), leading to long-range cation transfer^18^. After the tandem 1,2–H shifts (**IM5→IM7**) on the C-ring to yield a new allylic cation (the reaction pathway is discussed in detail below), a 1,5–H shift across the B-ring gives a homoallylic cation intermediate (**IM9**). As for Part C, DFT calculations of the C8–C9 bond rearrangement process were essentially consistent with our previously proposed reaction mechanism based on isotope tracing experiments and NMR spectroscopic investigations. After the conformational change of **IM9** into **IM10** with a very small activation barrier, the C7–C8 π bond of the homoallylic cation in **IM10** approaches the cation centre to form a cyclopropylcarbinyl cation intermediate (**IM11**) with an unexpectedly large stabilization energy (ΔG = –11.4 kcal/mol). **IM11** has a highly distorted cyclopropane ring in which participation of the elongated C8–C10 σ orbital partially contributes to stabilization of the neighboring C7 carbocation, as judged from the elongation of the C8–C10 bond (1.67 Å) as well as the short C7–C8 bond (1.38 Å)^19^. An unusual C–C bond rearrangement then takes place *via* a unique bicyclobutonium cation^20^ transition state (**TS_11-12**) with very low activation energy (6.5 kcal/mol) to give another cyclopropylcarbinyl cation (**IM12**). **IM12** is energetically more stable than **IM11** by 4.3 kcal/mol owing to the strain magnitudes of their cyclopropane rings. Finally, a hydration reaction yields the final product (**PD**). Although we should keep in mind the possibility that the CotB2 may be involved in the final step, our preliminary calculations indicate that non-enzymatic hydration of **IM12** with water molecules would also be likely to proceed smoothly to provide **PD**.

The energy diagram (Fig. 3) immediately suggests that this is a thermodynamically and kinetically favorable biosynthetic reaction cascade: 1) activation barriers are all low enough for the reactions to proceed smoothly at ambient temperature, 2) the entire energy profile descends as the reactions proceed, and 3) the overall exothermicity is very large^21^. The first annulation and conformational change (**IM2→TS_2-3**) is the rate-determining step. Remarkably, the cyclopropane ring formation and carbon–carbon bond rearrangement each require much lower activation energy than this step.

We next studied in detail the 1,3-cation shift reaction mechanism (**IM5→IM7**), which is the last puzzle in the biosynthesis. Two pathways are possible, i.e., direct 1,3-hydrogen shift and sequential double 1,2-hydrogen shift (Fig. 4). We would expect *a priori* that the concerted 1,3-H shift is a reasonable route, because of the stereoselectivity and step-economy, and so it was found. The 1,3-H shift from the C6 to the C3 position takes place as a single event with an overall energy loss of 20.7 kcal/mol. We could also identify the TSs for the two successive non-concerted 1,2-H shifts: the first 1,2-H shift from the C2 to C3 *via* **TS_5-6** yielding **IM6** followed by the second one from C6 to C2 *via* **TS_6-7** generating an allyl cation **IM7**. The activation energies for the first and second 1,2-H shifts are 7.1 kcal/mol and only 1.5 kcal/mol, respectively. The more than 10 kcal/mol difference of energy between the 1,3- and the tandem 1,2-transfer translates into an over 10^10^-fold difference of reaction rate at room temperature; hence the sequential double 1,2-hydrogen shift should predominate over direct 1,3-hydrogen shift in the microorganism.

To validate the successive 1,2-hydrogen migrations suggested by our calculation, we next incubated recombinant CotB2^6^ individually with two regiospecifically deuterated GGDPs, **2D-** and **6D-GGDP** (which were successfully prepared with >99% D incorporation ratio *via* the multistep procedure shown in Fig. 5 and characterized by means of high-resolution mass analysis and NMR spectroscopy as shown in the SI). Comparison of the deuterated product generated in each reaction by means of ^1^H NMR spectroscopy with natural abundance **2** (Fig. S1–S2) allowed assignment of the positions of deuteration in each product, thereby establishing that **2D-** and **6D-GGDP** were converted to **3D-** and **2D-PD,** respectively (Fig. 6). The formation of **3D-** and **2D-PD** from **2D-** and **6D-GGDP**, respectively, is fully consistent with the occurrence of successive 1,2-hydrogen migrations from C2 to C3 and C3 to C6 during the cyclization reaction cascade.

In summary, our computational and experimental studies have clarified the whole biosynthetic route to cyclooctatin. DFT calculations reveal that the core framework of cyclooctatin is constructed via tandem carbocation transfer (“cation-stitching cascade”), involving several hydrogen-shifts as well as cyclopropylcarbinyl cation rearrangement. Such a reaction cascade provides high efficiency and selectivity, and is well supported by the results of the deuteration experiments. Thus, the combination of theoretical calculations and experimental studies has provided detailed insight into this biosynthetic mechanism. This approach may also throw light on the evolution of particular biosynthetic pathways, and provide clues for designing new synthetic routes for complex functional molecules.

## Methods

All calculations were performed with the Gaussian 09 program^12^. Geometry optimizations were performed in the gas phase at the M062X/6-31G** level^11^, without any symmetry restrictions, and the nature of the minima was verified by frequency calculations within the harmonic approximation. IRC for all TS was performed with GRRM11^13^ and/or Reaction Plus^14^ programs based on Gaussian 09. Gibbs free energy was used as the basis for discussion in this study. The Cartesian coordinates of all species are collected in Supporting Information. Details for the synthesis of deuterated GGDPs and the corresponding deuterium tracing experiments are described in Supporting Information as well.

## Additional Information

**How to cite this article**: Sato, H. *et al.* "Cation-Stitching Cascade": exquisite control of terpene cyclization in cyclooctatin biosynthesis. *Sci. Rep.* **5**, 18471; doi: 10.1038/srep18471 (2015).

## Supplementary Material

Supplementary Information

## Figures and Tables

**Figure 1:**
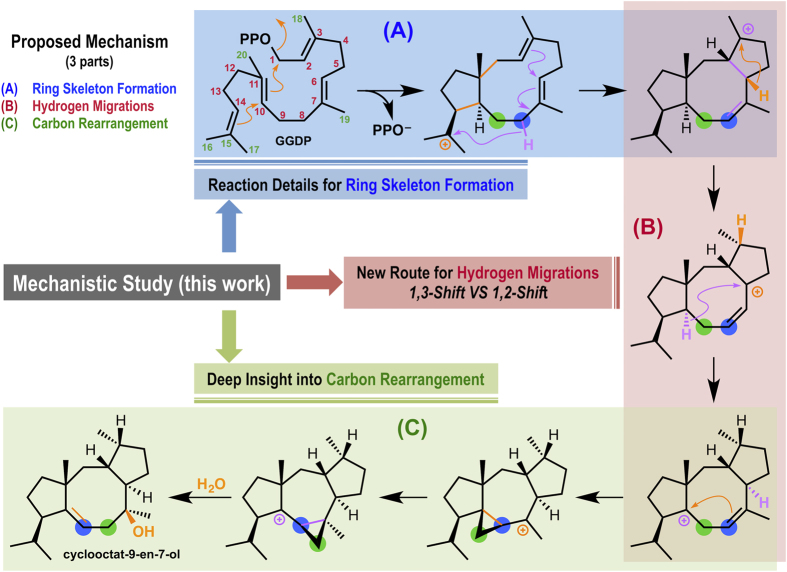
Proposed biosynthetic route to cyclooctat-9-en-7-ol. Putative carbocations involved in the conversion of geranylgeranyl diphosphate (GGDP) to cyclooctat-9-en-7-ol.

**Figure 2:**
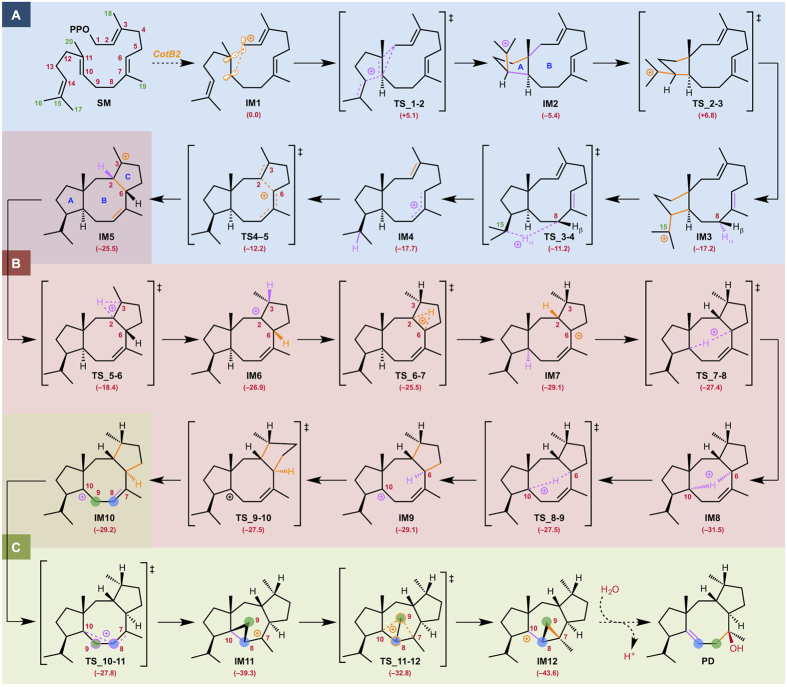
Computed reaction pathways and potential energy changes for the “cation-stitching cascade” from SM to PD. Potential energies (kcal/mol, Gibbs free energies calculated at the M062X/6-31G** level) relative to **IM1** are shown in parentheses.

**Figure 3:**
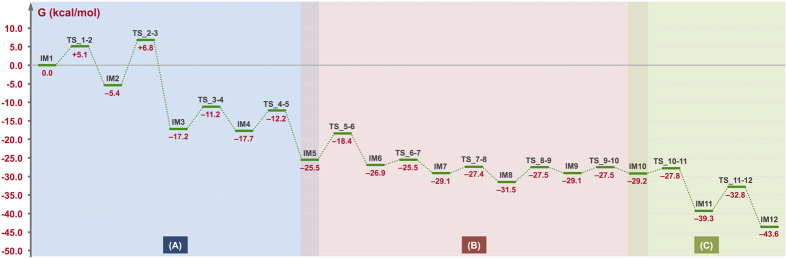
Computed potential energy profiles for the “cation-stitching cascade” reaction. Potential energies (kcal/mol, Gibbs free energies calculated at the M062X/6-31G** level) relative to **IM1** are shown in red.

**Figure 4:**
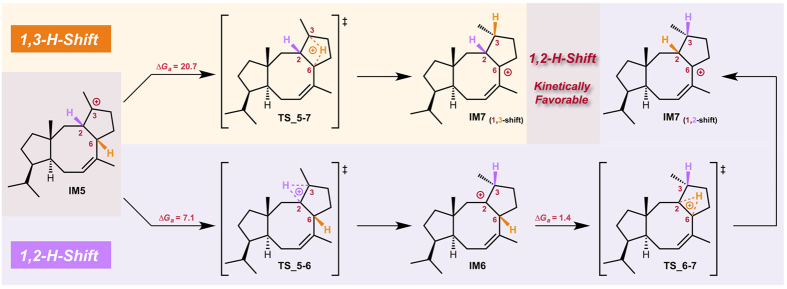
Comparison of the two possible hydrogen shift pathways explored theoretically. Energy changes (kcal/mol, Gibbs free energies calculated at the M062X/6-31G** level) are shown together with arrows.

**Figure 5:**
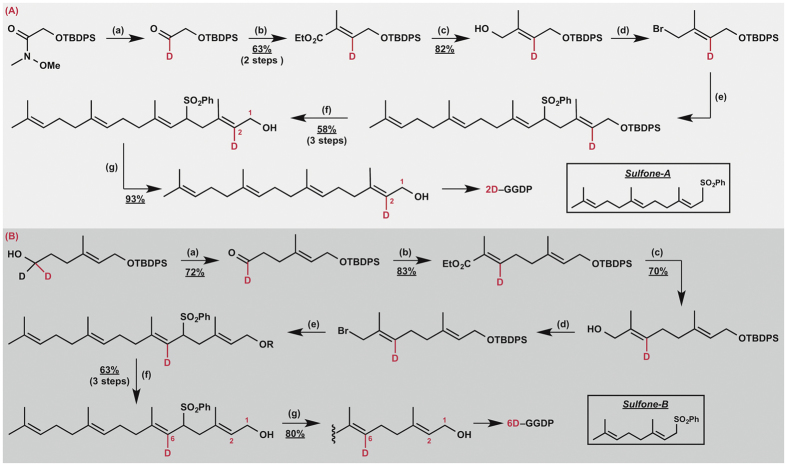
Regioselective synthesis of the deuterated GGDPs. (**A**) Reaction conditions: **(a)** LiAlD_4_ (1.0 equiv.), Et_2_O, −60 °C, 0.5 h; **(b)** EtO_2_C–C(CH_3_) = PPh_3_ (1.2 equiv.), CH_2_Cl_2_, rt, 1 h; **(c)** LiAlH_4_ (1.5 equiv.), Et_2_O, −40 to −20 °C, 1 h; **(d)** CBr_4_ (1.3 equiv.), PPh_3_ (1.3 equiv.), CH_2_Cl_2_ rt, 0.5 h; **(e)** *Sulfone-A* (1.5 equiv.), *t*-BuOK (4.0 equiv.), THF, −20 °C, 1 h; **(f)** TBAF (4.0 equiv.), THF, rt, 3 h; **(g)** LiBHEt_3_ (5.0 equiv.), Pd(dppp)Cl_2_ (5.0 mol%), THF, 0 °C, 1 h. **(B)** Reaction conditions: **(a)** PCC (2.0 equiv.), CH_2_Cl_2_, rt, 3 h; **(b)** EtO_2_C–C(CH_3_) = PPh_3_ (1.2 equiv.), CH_2_Cl_2_, rt, 2 h; **(c)** LiAlH_4_ (1.5 equiv.), Et_2_O, −40 to −20 °C, 1 h; **(d)** CBr_4_ (1.3 equiv.), PPh_3_ (1.3 equiv.), CH_2_Cl_2_ rt, 0.5 h; **(e)** *Sulfone-B* (1.5 equiv.), *t*-BuOK (4.0 equiv.), THF, −20 °C, 1 h; **(f)** TBAF (4.0 equiv.), THF, rt, 3 h; **(g)** LiBHEt_3_ (5.0 equiv.), Pd(dppp)Cl_2_ (5.0 mol%), THF, 0 °C, 2 h.

**Figure 6:**
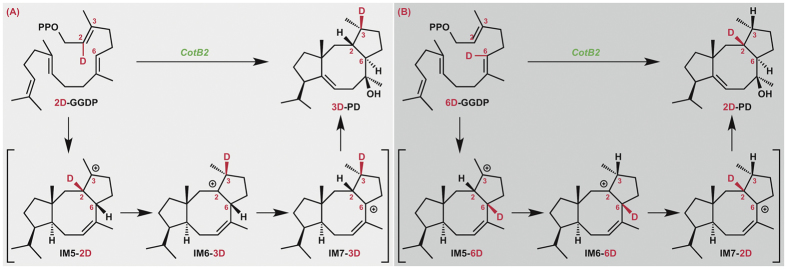
Isotope tracing experiments for the biosynthesis of cyclooctatin.
