# Supplementary material for: “Cation-Stitching Cascade”: exquisite control of terpene cyclization in cyclooctatin biosynthesis
Source: Sci Rep. 2015 Dec 18;5:18471. doi: 10.1038/srep18471 (PMC4683443; doi:10.1038/srep18471)
Supplement: Supplementary Information [file srep18471-s1.pdf]

SUPPORTING INFORMATION

"Cation-Stitching Cascade": an exquisite control of terpene cyclization in cyclooctatin biosynthesis

Hajime Sato,[1,2] Kazuya Teramoto,[3] Yui Masumoto,[1,2] Noriyuki Tezuka,[1,2] Kenta Sakai,[4] Shota Ueda,[4] Yusuke Totsuka,[4] Tetsuro Shinada,[4] Makoto Nishiyama,[3] Chao Wang,[1,2] Tomohisa Kuzuyama,*[3] and Masanobu Uchiyama*[1,2]

[1] Graduate School of Pharmaceutical Sciences, The University of Tokyo, 7-3-1 Hongo, Bunkyo-ku, Tokyo 113-0033, Japan;
[2] Elements Chemistry Laboratory, RIKEN, and RIKEN Center for Sustainable Resource Science (Wako campus), 2-1 Hirosawa, Wako-shi, Saitama-ken 351-0198, Japan.
[3] Biotechnology Research Center, The University of Tokyo, 1-1-1 Yayoi, Bunkyo-ku, Tokyo 113-8657, Japan.
[4] Department of Material Science, Graduate School of Science, Osaka City University, Sugimoto, Sumiyoshi-ku, Osaka 558-8585, Japan.

E-mail: utkuz@mail.ecc.u-tokyo.ac.jp (T.K.); uchiyama@mol.f.u-tokyo.ac.jp (M.U.)

Table of Contents

1.	Calculation Methods	S-2
2.	Energy Profiles	S-2
3.	Cartesian Coordinates	S-3
4.	Experiment Details	S-16
5.	Reference	S-24

1. Calculation Methods

All calculations were performed with Gaussian 09 program.[1] Geometry optimizations were performed in the gas phase at the M062X/6-31G** level,[2-3] without any symmetry restrictions, and the nature of the minima was verified by frequency calculations within the harmonic approximation. IRC for all TS was performed with GRRM11[4] and/or Reaction Plus[5-7] programs based on Gaussian 09. Gibbs free energy used for discussion in this study.

2. Energy Profiles

	G(a.u.)
**INT1**	-780.891409
**TS1-2**	-780.883222
**INT2**	-780.899979
**TS2-3**	-780.880606
**INT3**	-780.918933
**TS3-4**	-780.909235
**INT4**	-780.919727
**TS4-5**	-780.910952
**INT5**	-780.932079
**TS5-6**	-780.920758
**INT6**	-780.934369
**TS6-7**	-780.932036
**INT7**	-780.937756
**TS7-8**	-780.935104
**INT8**	-780.941615
**TS8-9**	-780.935329
**INT9**	-780.937778
**TS9-10**	-780.935275
**INT10**	-780.937947
**TS10-11**	-780.935784
**INT11**	-780.954009
**TS11-12**	-780.943667
**INT12**	-780.960904
**1,3-shift-Reverse**	-780.924721
**1,3-shift-TS**	-780.89807
**1,3-shift-Forward**	-780.948381

3. Cartesian Coordinates

INT1

C	-2.02039648	1.56258457	0.43101538
C	-2.74658985	0.89016243	1.33223888
C	-1.76921239	-1.36514886	-0.41134891
H	-2.26943497	0.61352394	2.27538171
H	-0.18736795	-2.02628163	-1.73703715
H	-0.94455861	-0.50966376	-2.21200792
C	-0.55735673	1.85381302	0.64829932
H	-0.25327258	1.56489069	1.65715936
C	0.37892627	1.26371143	-0.43476969
H	0.08761430	1.69200243	-1.40028149
H	1.38917928	1.64899258	-0.24439650
C	1.99037642	-2.21056701	0.00482020
H	1.79305361	-3.09338764	0.61852299
H	1.95850796	-2.45761578	-1.05871984
C	3.44796287	-1.68701073	0.35581792
H	4.09860198	-2.53643792	0.12729039
H	3.51261901	-1.51212755	1.43134885
H	3.94893792	-0.66799932	-1.52147665
C	-3.08071757	-1.20972833	-0.63553999
H	-1.49560264	-1.65013900	0.60293916
C	-3.99234164	-1.16916862	0.56329362
H	-4.99540954	-1.53656774	0.32678125
H	-3.59080509	-1.79613360	1.36609969
C	-4.10642025	0.30295077	1.08810532
H	-4.66680530	0.89426354	0.35893771
H	-4.69945002	0.28879611	2.00649134
C	-3.71183037	-0.91985602	-1.96642654
H	-2.98897134	-0.74313718	-2.76563190
H	-4.32807404	-1.77507097	-2.26488370
H	-4.38137879	-0.05581918	-1.90498502
C	-2.55136290	2.04111430	-0.89543692
H	-2.15987904	3.03903073	-1.12105056
H	-2.24208389	1.38378578	-1.71721557
H	-3.63945796	2.10065482	-0.91778695
C	4.08944875	0.75109950	-0.00072944
H	-0.39271576	2.93774637	0.58869375
C	4.49071494	1.84603428	-0.95253372
H	3.80895308	2.70012384	-0.86909035
H	5.48998820	2.21825731	-0.70421401
H	4.49879937	1.50589967	-1.98945369
C	4.02175744	1.18144305	1.44124516
H	3.79831634	0.37330274	2.13997034
H	4.97850390	1.61952294	1.74257476
H	3.27148306	1.97193668	1.56764031
H	1.32319641	-0.30166164	-1.52494380
C	0.81041036	-0.84691200	1.76099914
H	-0.23939578	-0.56689899	1.89684528
H	1.10328386	-1.66442592	2.41941685
H	1.38359900	0.05936193	2.02240227
C	3.84281544	-0.48660644	-0.45169541
C	0.58326819	-0.24654275	-0.71467165
C	1.09580321	-1.10464109	0.34480774
C	-0.63416728	-1.10441003	-1.34763433

TS1-2

C	-1.92690000	1.74336700	0.47511300
C	-2.64576700	0.90254000	1.22497900
C	-1.72984300	-1.76825100	-0.14559600
H	-2.11688800	0.32149100	1.98260300
H	-0.00790800	-2.82172900	-0.94927700
H	-0.54250200	-1.47055900	-1.93030800
C	-0.41497700	1.74846300	0.55380600
H	-0.05794000	1.30081100	1.48312300
C	0.21841300	1.07169800	-0.69062600
H	-0.45035800	1.17564100	-1.55430100
H	1.11389100	1.64180900	-0.98255100
C	2.16456800	-2.27061500	-0.05800700
H	2.00005900	-3.21196700	0.47506500
H	2.22520600	-2.49210500	-1.12944400
C	3.50207600	-1.62688100	0.38719000
H	4.27935000	-2.38669700	0.25992200
H	3.46765100	-1.39865800	1.45554700
H	4.16209100	-0.63551600	-1.45804300
C	-2.86586300	-1.16441100	-0.53845400
H	-1.72719400	-2.25467100	0.82574500
C	-3.99803200	-1.02272200	0.45031000
H	-4.95863600	-1.28053500	-0.00677200
H	-3.83694100	-1.70217700	1.29224800
C	-4.05445900	0.44740600	0.98071000
H	-4.56066300	1.08273000	0.25135600
H	-4.65806100	0.46124700	1.89236200
C	-3.09171000	-0.51259600	-1.86940400
H	-2.21260800	-0.48753400	-2.51597300
H	-3.88218800	-1.05127100	-2.40313800
H	-3.44624300	0.51329700	-1.73410100
C	-2.49935900	2.70343100	-0.53635200
H	-2.24543200	3.72769700	-0.24155900
H	-2.08354700	2.55793000	-1.54043200
H	-3.58537000	2.64803500	-0.61177400
C	3.76959000	0.87044800	-0.07536400
H	-0.05284200	2.78317000	0.55503600
C	4.13840800	1.96616300	-1.04044500
H	3.32757600	2.69969100	-1.13212500
H	5.01031600	2.51848500	-0.67430900
H	4.37212100	1.57795600	-2.03353700
C	3.35515400	1.35992900	1.28702700
H	3.21799400	0.56166400	2.01778700
H	4.10938600	2.04624000	1.68488900
H	2.42195400	1.93488600	1.22166600
H	1.34579400	-0.47688100	-1.65745600
C	0.56372400	-1.25068600	1.64424800
H	-0.37285300	-0.71411100	1.76168700
H	0.46033300	-2.25829700	2.05489400
H	1.33421200	-0.73304800	2.22514000
C	3.83774700	-0.41753800	-0.43953200
C	0.78646700	-0.32377400	-0.73439800
C	1.02448400	-1.31370900	0.21669400
C	-0.47534800	-1.83843100	-0.90589400

INT2

C	-1.58316156	1.90599377	0.31034679
C	-2.36541413	1.12916254	1.06500346
C	-1.67337118	-1.87995274	0.11704276
H	-1.87801561	0.52818236	1.83517704
H	-0.24761424	-3.37638168	-0.36559683
H	-0.41873830	-2.17455437	-1.62880454
C	-0.08054264	1.70321947	0.35780762
H	0.18770841	1.18969798	1.28429097
C	0.28962686	0.85352888	-0.88878916
H	-0.63789466	0.40799346	-1.26739543
H	0.62964227	1.50965188	-1.69922289
C	2.14788117	-2.45447198	-0.25258058
H	2.20768888	-3.32459500	0.40605612
H	2.12099130	-2.82447824	-1.28427667
C	3.33972966	-1.50373222	-0.05827114
H	4.22351929	-1.81913693	-0.61621706
H	3.62855456	-1.46927563	0.99388435
H	3.22428951	0.10433575	-1.56972746
C	-2.72424265	-1.21898616	-0.38558758
H	-1.74051611	-2.15488058	1.16926249
C	-3.83933390	-0.78182479	0.54372964
H	-4.81905335	-1.04181309	0.12796799
H	-3.74051847	-1.31171295	1.49641998
C	-3.79612768	0.75179685	0.81935171
H	-4.20746003	1.29600708	-0.03436188
H	-4.43796489	0.96885929	1.67896832
C	-2.86814866	-0.76777431	-1.81203223
H	-2.04957617	-1.09612800	-2.45594078
H	-3.80149317	-1.15238393	-2.23584940
H	-2.92836681	0.32733535	-1.86196342
C	-2.06183646	2.84001440	-0.76882837
H	-1.69243232	3.85294132	-0.57213557
H	-1.68392001	2.55282685	-1.75783833
H	-3.14910047	2.89419874	-0.82499701
C	3.07354212	1.06561424	0.23125176
H	0.45524424	2.66144909	0.33653157
C	3.14868206	2.36410556	-0.46941787
H	2.86073401	3.20811118	0.15900703
H	4.21968975	2.48786631	-0.70658768
H	2.61703031	2.36546005	-1.42217069
C	3.25482163	1.11317836	1.69455048
H	3.47854922	0.15821832	2.16195329
H	4.02575267	1.85254947	1.93855956
H	2.32381917	1.51580481	2.12173314
H	1.28574220	-0.70748655	-1.84398173
C	0.76523328	-1.38264597	1.53161212
H	-0.09204289	-0.76110631	1.78560692
H	0.62609974	-2.34962060	2.02573195
H	1.65970364	-0.92919882	1.96438183
C	2.87567139	-0.12040981	-0.55657513
C	1.22762790	-0.34205941	-0.81245222
C	0.89699957	-1.60347208	0.02237240
C	-0.38608535	-2.30124895	-0.54070044

TS2-3

C	-1.76008954	1.70975164	-0.15818437
C	-2.59840918	1.57871756	0.87483447
C	1.19753871	0.00655621	-0.84150248
C	-1.75048911	-1.30276263	0.59460652
C	0.83833688	-1.10668495	0.18930135
C	-0.52614927	-1.83416019	-0.08393172
H	-2.20856677	1.76091446	1.87811871
H	-0.37676777	-2.85815488	0.28470798
H	-0.66486879	-1.92737863	-1.16831909
C	-0.32328134	2.13780122	0.04056016
H	-0.03239164	2.05029149	1.09164704
C	0.70376895	1.48231599	-0.90796106
H	0.32443312	1.62070925	-1.92382224
H	1.57942012	2.12972455	-0.87828303
C	1.95115751	-2.18388263	-0.06230082
H	2.36185759	-2.51823143	0.89619119
H	1.52629625	-3.07411417	-0.52943175
C	3.03321142	-1.59600653	-0.98624267
H	2.85619103	-1.89909261	-2.02093070
H	4.04525604	-1.92287960	-0.73576271
C	2.84967930	-0.08150460	-0.91963173
H	3.07654639	0.46040341	-1.84657197
C	-3.00705529	-1.21134786	0.14830550
H	-1.59843461	-0.99117036	1.62566549
C	-4.00616412	-0.51107704	1.03852257
H	-5.02220340	-0.88324614	0.87173693
H	-3.76011072	-0.69944422	2.08826813
C	-3.99382433	1.03067345	0.79753334
H	-4.44953573	1.24372998	-0.17292798
H	-4.63299089	1.49935504	1.55132814
C	-3.49586062	-1.64061890	-1.20634418
H	-2.69076283	-1.93794749	-1.88179704
H	-4.17688511	-2.49271402	-1.10486722
H	-4.06746176	-0.83891880	-1.68711563
C	-2.15832822	1.47883496	-1.59205218
H	-1.79334412	2.28762321	-2.23594658
H	-1.73281581	0.53925586	-1.97115060
H	-3.23742736	1.41989803	-1.72330212
C	3.56334019	0.59072018	0.14973032
H	-0.25571112	3.20827795	-0.20201650
C	3.72945247	2.05672759	0.17646993
H	2.91141758	2.43598902	0.81601841
H	4.65897520	2.34335708	0.67515868
H	3.64397430	2.53255990	-0.79887732
C	4.26732029	-0.14637004	1.21502354
H	3.87939688	-1.14291094	1.41051608
H	5.29367489	-0.25966712	0.81855124
H	4.36048934	0.44181278	2.13096278
H	0.98244851	-0.43679044	-1.82092756
C	0.89729178	-0.58255788	1.62574044
H	0.08911786	0.11969949	1.82849329
H	0.81581421	-1.40649385	2.34204885
H	1.83855023	-0.06257640	1.83831859

INT3

C	-1.31107134	2.02291818	0.45353973
C	-2.42554740	1.32688257	0.70618206
C	0.77876539	-0.04877880	0.21211163
C	-1.73989650	-1.84184454	0.38893731
C	0.62584853	-1.29920828	1.13217769
C	-0.31403266	-2.32174697	0.42340230
H	-2.49734369	0.84147124	1.68162272
H	-0.25250938	-3.26540481	0.97839666
H	0.05368473	-2.53630483	-0.58636739
C	-0.16989621	2.01619016	1.44766244
H	-0.49411060	1.55310688	2.38019118
C	1.09329180	1.30134739	0.89142406
H	1.53556533	1.99794169	0.16913040
H	1.83359848	1.18453810	1.69167080
C	2.05082816	-1.85672544	1.24914811
H	2.63753735	-1.20457274	1.90925229
H	2.06467799	-2.85631843	1.69158173
C	2.62209709	-1.82847793	-0.16788307
H	2.39174445	-2.75070742	-0.70586913
H	3.70947193	-1.72335761	-0.18493398
C	1.90382391	-0.67420878	-0.88866122
H	1.27309956	-1.00544716	-1.71665439
C	-2.37750829	-1.20076751	-0.59856558
H	-2.28384104	-1.96041926	1.32507801
C	-3.71172605	-0.54539950	-0.32703236
H	-4.43678901	-0.77823190	-1.11456091
H	-4.11885377	-0.92577918	0.61509038
C	-3.55886174	0.99769623	-0.22207127
H	-3.39715527	1.42258447	-1.21635172
H	-4.50132797	1.41348312	0.15010379
C	-1.81147863	-0.94532546	-1.97080120
H	-0.90855208	-1.52631560	-2.17555938
H	-2.54845250	-1.20025509	-2.73885229
H	-1.58058409	0.12194703	-2.10372798
C	-1.05535275	2.79585826	-0.81380311
H	-0.77510418	3.82783932	-0.57450258
H	-0.21933382	2.37122132	-1.38585420
H	-1.92280455	2.83289588	-1.47272810
C	2.63052383	0.49600874	-1.25200028
H	0.13334301	3.04126694	1.69351097
C	2.15688090	1.33550554	-2.36861802
H	2.24078207	2.40384257	-2.15085637
H	2.84910142	1.13649102	-3.20359800
H	1.15113357	1.07094886	-2.69828779
C	3.87110155	0.88763470	-0.55484954
H	4.01735200	1.96890839	-0.54084644
H	3.94099138	0.46317107	0.44747785
H	4.69529241	0.45576314	-1.14663659
H	-0.11448792	0.09544029	-0.39759617
C	0.08659284	-0.98884826	2.53225422
H	-0.91718840	-0.56577483	2.50399177
H	0.04041250	-1.92319930	3.10093447
H	0.74275105	-0.30487843	3.07728609

TS3-4

C	0.92741300	2.00584700	-0.88093700
C	1.92938100	1.09470900	-0.97143700
C	-0.64112700	-0.55466800	-0.25854700
C	1.83861500	-2.01630600	0.22837300
C	-0.65733500	-2.10484200	-0.27242400
C	0.51001500	-2.59747900	0.64380700
H	1.71947400	0.16731700	-1.49900500
H	0.53882400	-3.69273300	0.61018700
H	0.27502000	-2.32227600	1.67698700
C	-0.39039600	1.62453700	-1.37489500
H	-0.97934600	2.40100800	-1.87141000
C	-0.74295100	0.19183000	-1.61110100
H	-1.73290000	0.10505800	-2.06617500
H	-0.03173000	-0.23045500	-2.32953000
C	-2.05376300	-2.46982300	0.32550800
H	-2.53735800	-3.27967200	-0.22607700
H	-1.94057200	-2.80963200	1.36100000
C	-2.88331800	-1.17205500	0.30339100
H	-3.71872200	-1.19011800	1.00784900
H	-3.29802200	-0.99891700	-0.69716400
C	-1.81503400	-0.12501400	0.63954500
H	-1.49110900	-0.30981600	1.67398400
C	2.48994300	-0.98875500	0.79881600
H	2.26134400	-2.41968400	-0.69271500
C	3.65754200	-0.32593300	0.09188600
H	4.53735000	-0.27047400	0.74136600
H	3.93636100	-0.91349000	-0.78838100
C	3.29399800	1.12005100	-0.36304600
H	3.32758900	1.81684100	0.47930000
H	4.03769900	1.46374300	-1.09294700
C	2.07803700	-0.31713600	2.08467200
H	2.94447900	-0.20130900	2.74369100
H	1.69218600	0.69531400	1.89487500
H	1.31416200	-0.86925100	2.63272300
C	1.07792300	3.38389200	-0.28952800
H	2.12521200	3.64712300	-0.14367200
H	0.63502600	4.13409200	-0.95162100
H	0.56817200	3.45766900	0.67754900
C	-2.10353500	1.37393300	0.53328400
H	-1.21176900	1.78614700	-0.18377300
C	-1.84421600	2.14937500	1.81274600
H	-1.91854200	3.22813400	1.65859600
H	-2.60305700	1.86087400	2.54960500
H	-0.86453800	1.90509700	2.23378300
C	-3.37700900	1.77774500	-0.17886400
H	-3.46480500	1.33026700	-1.17203400
H	-4.22870900	1.42175100	0.41197700
H	-3.46034100	2.86348500	-0.26492500
H	0.28500000	-0.21908200	0.19615600
C	-0.50922600	-2.70629100	-1.67366700
H	0.43179900	-2.41903100	-2.15427600
H	-0.52550600	-3.79787900	-1.61262000
H	-1.33241100	-2.40178800	-2.32877800

INT4

C	1.22049311	1.98113121	-1.10500381
C	2.13713152	0.94336174	-1.17583885
C	-0.71676176	-0.37914461	-0.10645011
C	1.84607620	-1.94269390	0.22043998
C	-0.66354337	-1.95208091	-0.19375955
C	0.51230931	-2.42798187	0.71103991
H	1.83112400	0.01415783	-1.65208753
H	0.50021848	-3.52469907	0.73107553
H	0.31890347	-2.09258637	1.73465386
C	-0.12403996	1.67561910	-1.34973031
H	-0.83199412	2.50183916	-1.26179073
C	-0.68030590	0.33043780	-1.52599874
H	-1.67680870	0.36021488	-1.96708419
H	-0.03731799	-0.26522299	-2.17590679
C	-2.03782133	-2.41402800	0.38010807
H	-2.44373035	-3.26037867	-0.17933656
H	-1.91869973	-2.73908229	1.41989869
C	-2.93699413	-1.17565407	0.33883985
H	-3.80741686	-1.25719593	0.99489738
H	-3.30735375	-1.00017166	-0.68018289
C	-1.95968037	-0.07427717	0.75396471
H	-1.63478869	-0.33151827	1.77471641
C	2.61242315	-0.96495004	0.73520053
H	2.20184207	-2.41202768	-0.69786538
C	3.81554527	-0.46460514	-0.04634515
H	4.71004272	-0.40132614	0.58061279
H	4.03728083	-1.15051354	-0.86920483
C	3.51002736	0.94234689	-0.62146593
H	3.61879584	1.72701073	0.13286865
H	4.21465159	1.19091462	-1.43156332
C	2.34408090	-0.23740028	2.02615106
H	3.20099037	-0.34531928	2.69928392
H	2.21059430	0.83994092	1.86054896
H	1.46015695	-0.60391299	2.54791239
C	1.62594404	3.36000707	-0.63977173
H	2.53112283	3.70040166	-1.14620485
H	0.83375109	4.08253982	-0.84210641
H	1.81709680	3.36811381	0.43808063
C	-2.47843124	1.37501570	0.83766203
H	-1.59738745	2.03965973	0.82782304
C	-3.18411160	1.59509739	2.18072258
H	-3.55214065	2.62042329	2.26719640
H	-4.04485727	0.92480526	2.27446317
H	-2.51272963	1.40207461	3.02159170
C	-3.43090711	1.78616212	-0.29061656
H	-3.00821398	1.70054069	-1.29567049
H	-4.33497531	1.17014292	-0.26710885
H	-3.74339562	2.82528724	-0.15660806
H	0.17714565	-0.02761145	0.41314971
C	-0.50201653	-2.53042896	-1.60291918
H	0.42989833	-2.23339332	-2.09519360
H	-0.49636962	-3.62242550	-1.54553980
H	-1.33710581	-2.24250415	-2.25027186

TS4-5

C	1.79860600	1.61370600	-0.64455700
C	2.32754500	0.35126900	-1.03820200
C	-1.00495300	-0.10511700	-0.02161600
C	1.88615700	-1.56989500	0.34179900
C	-0.65595400	-1.64509700	-0.20619300
C	0.51864400	-2.05728100	0.71521800
H	1.68788400	-0.24323000	-1.67932900
H	0.56660200	-3.15488500	0.71245900
H	0.26663800	-1.75510900	1.73513900
C	0.45206900	1.81516300	-0.78261600
H	0.08208300	2.79488300	-0.48011500
C	-0.57868700	0.82700400	-1.21637500
H	-1.44064200	1.38321800	-1.58442000
H	-0.21711500	0.23515600	-2.05834800
C	-1.94179700	-2.36579200	0.26861100
H	-2.02939800	-3.36593200	-0.16442900
H	-1.92556000	-2.48061000	1.35964800
C	-3.07237300	-1.42041900	-0.10859700
H	-4.01583900	-1.68621300	0.37201800
H	-3.24361200	-1.41343700	-1.19353500
C	-2.53323900	-0.06780100	0.36182100
H	-2.55763200	-0.09534200	1.46234100
C	2.65130000	-0.67365800	1.03654300
H	2.35891900	-2.09058300	-0.49169500
C	4.03464200	-0.31133300	0.49816400
H	4.41364200	0.56685400	1.02306700
H	4.75654500	-1.11681400	0.65864300
C	3.79771500	0.00691200	-0.98812300
H	4.40853400	0.83955200	-1.34821700
H	3.99870700	-0.85373400	-1.62798900
C	2.18017200	0.02125400	2.27531500
H	3.02894300	0.30363400	2.90259500
H	1.65835800	0.95022900	1.99624800
H	1.48876900	-0.57772700	2.86806800
C	2.70210200	2.69008100	-0.09137500
H	3.51973300	2.91670400	-0.78113100
H	2.14114500	3.61140300	0.07183100
H	3.14644100	2.40040300	0.86510400
C	-3.37060300	1.15135900	-0.04277000
H	-3.32491500	1.27680000	-1.13339300
C	-2.86033700	2.42219800	0.63980100
H	-3.39886100	3.30526000	0.28716100
H	-3.01144800	2.34969300	1.72235200
H	-1.79247800	2.59238000	0.47421300
C	-4.84429800	0.93954700	0.31834900
H	-5.29649900	0.13192500	-0.26133700
H	-4.95065000	0.69855700	1.38211300
H	-5.41783300	1.84950200	0.12411100
H	-0.44426500	0.24208700	0.85350300
C	-0.39500100	-2.10245100	-1.64782500
H	0.51474800	-1.69824100	-2.10051600
H	-0.29112300	-3.19109200	-1.66496600
H	-1.23006300	-1.84319400	-2.30542900

INT5

C	1.82870196	1.59817478	-0.01625514
C	2.01565230	0.37623074	-0.88073777
C	-1.30411641	0.55108115	0.49144327
C	1.85159565	-0.91198570	-0.00586975
C	-0.72571585	-0.89389898	0.46150998
C	0.71314721	-1.04718396	1.00933404
H	1.25191398	0.37105808	-1.65702888
H	0.78727158	-2.04880154	1.45299333
H	0.87735697	-0.32559299	1.81947246
C	0.60879433	2.12707797	0.13129390
H	0.50691126	2.97320886	0.81025973
C	-0.67932155	1.60414291	-0.44632103
H	-1.37336032	2.44125666	-0.54803932
H	-0.54617867	1.20142012	-1.45561263
C	-1.69854488	-1.56874188	1.44827523
H	-1.63991053	-2.66152820	1.39994461
H	-1.43068396	-1.27116745	2.46972433
C	-3.08558081	-1.02608338	1.06856157
H	-3.73192071	-0.94322855	1.94448221
H	-3.58132707	-1.72020770	0.38957975
C	-2.85166094	0.36220995	0.39967270
H	-3.30724474	1.12676005	1.03869735
C	3.19957557	-1.26716461	0.40158787
H	1.75242200	-1.73426881	-0.76783737
C	4.20237445	-0.62164440	-0.46163389
H	4.77365467	0.03554500	0.21812968
H	4.94230713	-1.34333409	-0.83032787
C	3.40904963	0.17133227	-1.51148026
H	3.89529988	1.10886573	-1.78256040
H	3.31496631	-0.42530548	-2.42313597
C	3.53700303	-2.13159947	1.53554170
H	4.59209652	-2.39771352	1.57860623
H	3.25836241	-1.57655638	2.44627452
H	2.90185225	-3.02440495	1.54698520
C	2.99834759	2.08675414	0.79604653
H	3.88363574	2.29478716	0.18487472
H	2.74255326	3.00019705	1.33479600
H	3.29260694	1.34377719	1.55486028
C	-3.46108021	0.57977458	-1.02295192
H	-2.65804758	0.50345686	-1.76845875
C	-4.06520094	1.98684580	-1.12402535
H	-4.37918308	2.20905499	-2.14722628
H	-4.94858632	2.05681212	-0.47966061
H	-3.37146562	2.77021789	-0.80799643
C	-4.54263215	-0.42957116	-1.42133256
H	-4.15495210	-1.44098859	-1.57303297
H	-5.33174295	-0.48101613	-0.66360735
H	-5.00649719	-0.11952365	-2.36146956
H	-1.10965213	0.90241837	1.51601455
C	-0.86147605	-1.57636433	-0.90543145
H	-0.32781327	-1.05464752	-1.70709348
H	-0.48365400	-2.60536851	-0.85683438
H	-1.90791660	-1.63803138	-1.21220127

TS5-6

C	2.21984600	-1.65069100	-0.14927900
C	2.55084700	-0.18725600	-0.40683600
C	-0.66334500	-0.27534100	0.06078800
C	1.84953700	0.89866600	0.43844500
C	-0.28274700	0.05243300	1.54992000
C	1.17659900	0.59159400	1.75120700
H	3.61971000	-0.08705600	-0.17675600
H	1.78825000	-0.16056300	2.25641000
H	1.15076900	1.47624800	2.39613200
C	1.01592200	-2.24049800	-0.14582800
H	1.04986400	-3.32148400	-0.01250000
C	-0.39426900	-1.73153500	-0.31604200
H	-0.69010700	-1.91445000	-1.35607400
H	-1.04369500	-2.38515200	0.27615900
C	-1.33571600	1.10731400	1.94849200
H	-1.40643400	1.22355000	3.03428100
H	-1.07435800	2.08795900	1.52818200
C	-2.61522200	0.61077400	1.28265700
H	-3.40754400	1.36234300	1.27172600
H	-3.01049100	-0.26721900	1.80547000
C	-2.13784300	0.24764600	-0.13099900
H	-2.05712400	1.20644500	-0.66920600
C	1.62174400	2.03554800	-0.37011700
H	2.69667200	1.74740000	0.61772300
C	2.17257600	1.81744100	-1.75251300
H	1.48724600	2.25858200	-2.48344500
H	3.11933500	2.36326700	-1.85922000
C	2.33204400	0.29067900	-1.86264700
H	1.41359300	-0.15444900	-2.25708100
H	3.15118800	-0.00309600	-2.51951200
C	0.95425900	3.29868500	0.02763600
H	0.83209500	3.40136500	1.10606800
H	1.47094700	4.16629000	-0.38938900
H	-0.04519400	3.26764000	-0.42789100
C	3.46355200	-2.49083700	0.03206600
H	4.01788900	-2.18227000	0.92619900
H	3.22565300	-3.55049100	0.12915100
H	4.14282200	-2.37226100	-0.82024000
C	-3.08575000	-0.62108300	-0.97164200
H	-2.99146400	-1.66601300	-0.64758900
C	-2.73206000	-0.51992700	-2.46006200
H	-3.29905600	-1.24263500	-3.05204400
H	-2.98224200	0.48071400	-2.83051200
H	-1.66831100	-0.68465500	-2.65973200
C	-4.54542200	-0.20196200	-0.77745700
H	-4.88807200	-0.36030600	0.24779200
H	-4.67717600	0.85875200	-1.02064300
H	-5.19791400	-0.77672400	-1.43978800
H	-0.05490200	0.33630500	-0.61482100
C	-0.43870800	-1.15560600	2.48912100
H	0.27136900	-1.95035700	2.24949900
H	-0.25241300	-0.83788600	3.51972600
H	-1.44596500	-1.57605900	2.45046800

INT6

C	2.13236700	-1.49400600	-0.73899800
C	2.39999800	-0.07102400	-0.30363100
C	-0.70779500	-0.30726600	-0.01597400
C	1.71979800	0.61802300	0.79320200
C	-0.35992600	-0.60624900	1.49885000
C	1.06606100	-0.04667800	1.92146500
H	3.38776800	-0.07885200	0.23738200
H	1.69426200	-0.90207300	2.19987600
H	0.97328500	0.62783700	2.77611100
C	0.93908100	-2.03575000	-1.01579700
H	0.98531700	-3.05048500	-1.41041400
C	-0.46975500	-1.50846300	-0.92986700
H	-0.78640000	-1.26625300	-1.95108400
H	-1.11645300	-2.33942300	-0.62651400
C	-1.49201100	0.09345700	2.28115300
H	-1.60300800	-0.32312900	3.28613600
H	-1.29672300	1.16672600	2.39052000
C	-2.71087000	-0.09867200	1.38063900
H	-3.54977500	0.53372000	1.67822700
H	-3.06002500	-1.13787500	1.40153500
C	-2.16266300	0.27500500	-0.00194000
H	-2.05561800	1.37171700	0.01320700
C	1.91192200	2.08848700	0.66997100
H	2.34972200	2.43774000	1.61589600
C	2.82863600	2.27615300	-0.55864400
H	2.60007300	3.19770300	-1.09619200
H	3.87624600	2.32311900	-0.24589100
C	2.58731000	1.01157700	-1.39126800
H	1.66814800	1.07544300	-1.98504000
H	3.40635900	0.76981100	-2.06926900
C	0.55857000	2.83091800	0.51481800
H	-0.14986400	2.56511000	1.30156600
H	0.76047300	3.90097500	0.57781200
H	0.10604600	2.62169200	-0.45763300
C	3.40607900	-2.27111900	-0.97530000
H	3.95018300	-2.42964000	-0.03694400
H	3.20274500	-3.24715900	-1.41658800
H	4.07890500	-1.72927100	-1.64983100
C	-3.04799300	-0.04428500	-1.21312800
H	-3.00725000	-1.12389500	-1.40998900
C	-2.55169300	0.71544200	-2.44861100
H	-3.09273900	0.40793500	-3.34681500
H	-2.71848600	1.79015800	-2.31294000
H	-1.48205700	0.57176400	-2.63804200
C	-4.50845300	0.32507700	-0.94247100
H	-4.94259300	-0.27853800	-0.14173900
H	-4.59340400	1.38046500	-0.65887500
H	-5.11325400	0.17312800	-1.84004500
H	-0.06576600	0.49850400	-0.39798900
C	-0.39928700	-2.09838100	1.86038200
H	0.34579100	-2.68046700	1.31480200
H	-0.20451900	-2.21270000	2.93070700
H	-1.38364100	-2.52725900	1.65896200

TS6-7

C	-1.99644500	1.74661400	-0.25960000
C	-2.27797600	0.28631400	-0.31508300
C	0.78964600	0.24123600	0.04615500
C	-1.82710200	-0.71601000	0.57613900
C	0.43759100	0.01335800	1.56298400
C	-1.04101100	-0.45683900	1.81756400
H	-3.05209000	-0.00871500	0.61856700
H	-1.56333900	0.33340100	2.36911700
H	-1.05371400	-1.35305000	2.44425400
C	-0.76771300	2.28777800	-0.29918100
H	-0.76453500	3.37718900	-0.34203800
C	0.61372200	1.69674000	-0.38875600
H	0.93343400	1.82088600	-1.43054300
H	1.28222200	2.34871600	0.18342200
C	1.46217100	-1.04884900	2.01275200
H	1.57056400	-1.07231900	3.10119300
H	1.15000000	-2.05119000	1.69357200
C	2.73403600	-0.65905200	1.26473700
H	3.49121200	-1.44557500	1.28422400
H	3.18787000	0.23764400	1.70251500
C	2.21887000	-0.38369700	-0.15498700
H	2.05390700	-1.37497500	-0.60683700
C	-2.21947900	-2.08299500	0.06838700
H	-2.59908900	-2.69512800	0.89197600
C	-3.28518500	-1.78537700	-1.00661900
H	-3.29064100	-2.53679900	-1.79711400
H	-4.28157300	-1.77148200	-0.55262800
C	-2.93305800	-0.38039400	-1.51969400
H	-2.17306200	-0.40131400	-2.31009200
H	-3.78771400	0.17866600	-1.90143500
C	-0.97206900	-2.77270800	-0.51948700
H	-0.15626000	-2.83451300	0.20428700
H	-1.24656200	-3.78900600	-0.80786800
H	-0.60741900	-2.25435000	-1.41098600
C	-3.23980600	2.60774600	-0.28390000
H	-3.88841100	2.39738900	0.57414800
H	-2.97526200	3.66505800	-0.25108800
H	-3.83083900	2.43835600	-1.18949800
C	3.17842700	0.35560000	-1.09883000
H	3.17610100	1.42415800	-0.84330300
C	2.74045000	0.18188100	-2.55780700
H	3.33196300	0.81225400	-3.22643200
H	2.88994400	-0.85922200	-2.86497300
H	1.68388300	0.41850600	-2.72110000
C	4.61306000	-0.15592200	-0.94395300
H	5.01857500	0.04786700	0.04987800
H	4.65610300	-1.23776200	-1.11440400
H	5.26904200	0.32134300	-1.67650200
H	0.11935500	-0.35248200	-0.58278500
C	0.65815100	1.26952300	2.42335700
H	-0.02088500	2.08150300	2.14956600
H	0.47773400	1.02325700	3.47411500
H	1.68180300	1.64201900	2.34372200

INT7

C	-1.96898800	1.72514000	0.20801200
C	-2.19036600	0.37036400	-0.21716600
C	0.77438700	0.24009800	0.19586900
C	-1.94682500	-0.87417100	0.52153700
C	0.48129200	-0.48886500	1.54921700
C	-0.99410000	-0.94932300	1.71437900
H	-2.97009800	-0.81915200	0.99088600
H	-1.45111200	-0.34807800	2.50789100
H	-1.01876300	-1.98332200	2.07633300
C	-0.75808800	2.27080500	0.50267600
H	-0.79394900	3.33359100	0.74534900
C	0.62538700	1.75015600	0.30026600
H	0.96552600	2.24306800	-0.62266100
H	1.26972100	2.17490900	1.07750600
C	1.49593300	-1.65362700	1.55429800
H	1.66812700	-2.03178500	2.56633200
H	1.12937600	-2.49482400	0.95474500
C	2.73893200	-1.06145300	0.89450800
H	3.46350300	-1.82149200	0.59468000
H	3.25429600	-0.37385100	1.57578600
C	2.15718100	-0.30750700	-0.30968800
H	1.92231800	-1.07800600	-1.06092500
C	-2.13892300	-2.02574200	-0.50276500
H	-2.45892800	-2.92019300	0.03817600
C	-3.27511000	-1.46153300	-1.37446600
H	-3.32181700	-1.92194600	-2.36235400
H	-4.24425000	-1.61150200	-0.88804400
C	-2.96489000	0.04249700	-1.44961600
H	-2.25780300	0.26046900	-2.26902100
H	-3.81915500	0.70317500	-1.61688500
C	-0.89802600	-2.36294600	-1.32779500
H	-0.04477500	-2.61742700	-0.69601500
H	-1.11042100	-3.22611700	-1.96291500
H	-0.59613300	-1.53978900	-1.98536400
C	-3.19183100	2.63384100	0.11537100
H	-4.09321600	2.14465400	0.49208700
H	-3.02050100	3.52879700	0.71490200
H	-3.37599700	2.94772000	-0.91595100
C	3.08633500	0.69566200	-1.00663000
H	3.15067700	1.60605700	-0.39360100
C	2.54681400	1.05409000	-2.39671700
H	3.11893100	1.86892100	-2.84742000
H	2.63049800	0.18444600	-3.05779200
H	1.49159500	1.34913000	-2.38755300
C	4.50306500	0.13350900	-1.14946400
H	4.97890800	-0.03227600	-0.18021200
H	4.48468700	-0.82089400	-1.68777600
H	5.13227600	0.82343500	-1.71770800
H	0.03580500	-0.08217600	-0.55164200
C	0.79605300	0.39218900	2.77347000
H	0.13527700	1.26254800	2.83772000
H	0.64646900	-0.19541900	3.68392300
H	1.82954100	0.74822400	2.77575100

TS7-8

C	0.36391800	0.93823800	1.42429600
C	-1.87039800	-0.03983800	-0.48463500
C	-1.07967500	0.56456400	1.89808400
C	-1.93360400	-0.30550000	0.97021700
H	-1.62965900	1.49596000	2.06129200
H	-0.09289400	-0.24340600	-0.38070200
H	-1.03352100	0.06838000	2.87374000
H	-2.98910600	0.02985600	1.11212000
C	-0.79197000	2.04097200	-1.32388000
H	-0.94210900	2.99010700	-1.83426000
C	-1.87827500	1.28335100	-1.10682200
C	0.61751300	1.61036400	-1.07008600
H	1.67752200	0.83194200	3.18123100
H	1.19892400	-0.74511700	2.56346800
C	1.48174800	0.26914600	2.26444800
H	3.46389000	-0.47156700	1.67913200
H	3.12616800	1.18349400	1.16118200
C	2.02812500	-0.31607800	0.02506900
H	1.75764000	-1.36349500	0.22649200
C	-2.01673900	-1.84773300	1.10633900
C	4.25364100	-0.95820800	-0.97029000
H	4.14134600	-1.96388000	-0.54939200
H	4.82588700	-1.04676500	-1.89715500
H	4.84165600	-0.35991900	-0.27050600
C	2.88405800	-0.33199000	-1.24423300
H	-2.55812000	-2.09151500	2.02445100
C	-2.31910500	-1.26076000	-1.22485100
H	-3.01839500	-1.02406300	-2.03037500
H	-1.42334600	-1.66463000	-1.72230700
C	2.67744100	0.19400100	1.31761700
H	-2.71955500	-3.26935400	-0.42615500
H	-3.90307900	-2.03891800	0.02930000
C	-0.66718400	-2.55987500	1.12525500
H	-0.11575300	-2.42786700	0.18750400
H	-0.03591200	-2.21141500	1.94482200
H	-0.82168500	-3.63269800	1.26196900
C	-2.83758900	-2.22317400	-0.13994600
H	1.03887100	1.29829100	-2.03262600
H	1.21712000	2.47291600	-0.76211100
C	2.17162100	-1.11543400	-2.35267200
H	2.72567500	-1.06736500	-3.29324600
H	1.15592600	-0.75005700	-2.54824500
H	2.09369900	-2.17116700	-2.06774200
H	3.05225900	0.70155800	-1.57648700
C	0.69684100	0.49513400	-0.04246600
C	-3.25948800	1.69007800	-1.60438700
H	-3.25561600	2.76071800	-1.81276700
H	-3.52667900	1.16363800	-2.52441400
H	-4.03731300	1.49331200	-0.86078600
C	0.50681600	2.45948500	1.62889900
H	0.28601800	2.69457200	2.67372600
H	1.52344600	2.80631100	1.42387700
H	-0.18948700	3.03169400	1.00925000

INT8

C	0.32499600	-1.12269100	1.38854200
C	-1.44584700	0.54574500	-0.22905400
C	-1.18452200	-1.50611700	1.24750700
C	-1.97366400	-0.84223900	0.10599300
H	-1.67565300	-1.22890100	2.18396800
H	-0.21146400	0.38306000	-0.18924000
H	-1.26226400	-2.59616200	1.17337800
H	-2.97813900	-0.64354300	0.50905300
C	-0.69019800	2.02033900	1.57982600
H	-0.85959700	2.79926000	2.31694900
C	-1.67777000	1.62465000	0.77375400
C	0.70837200	1.49088500	1.41761300
H	1.53903700	-2.94285800	1.52157900
H	0.87164000	-2.65328200	-0.08287400
C	1.30583600	-2.16413100	0.79142200
H	3.17491200	-1.89081400	-0.33259800
H	3.12764200	-1.03363600	1.21327900
C	1.87478200	-0.12680200	-0.32032500
H	1.41803400	-0.51243800	-1.24734700
C	-2.22387500	-1.53355900	-1.25606500
C	4.01397400	0.52085000	-1.47523300
H	3.71963900	-0.08424800	-2.34016600
H	4.61128200	1.35839000	-1.84307300
H	4.65369200	-0.08821600	-0.83265900
C	2.78051400	1.03811300	-0.73058300
H	-3.05832500	-2.23299400	-1.14747000
C	-1.68792900	0.79979800	-1.71862500
H	-2.07104300	1.80559400	-1.90287700
H	-0.73849600	0.72201600	-2.26355800
C	2.52212200	-1.35034300	0.35489300
H	-2.58938400	-0.55277600	-3.20070800
H	-3.67217600	-0.04629900	-1.90040000
C	-1.04554800	-2.29796400	-1.84987600
H	-0.15724400	-1.66829800	-1.97402800
H	-0.77377800	-3.15692500	-1.23186300
H	-1.31174500	-2.68086500	-2.83813300
C	-2.64197400	-0.33696800	-2.13163100
H	1.32169400	2.24830400	0.92453900
H	1.16786300	1.32097400	2.39719800
C	2.01273600	2.02363600	-1.61887600
H	2.61232200	2.91199800	-1.83009600
H	1.06747900	2.36112900	-1.17568000
H	1.77796600	1.54970900	-2.57922700
H	3.13052900	1.55060200	0.17525300
C	0.72696900	0.18560100	0.65977000
C	-3.07059100	2.20010100	0.80965600
H	-3.13943700	2.97684600	1.57168400
H	-3.34305200	2.64222700	-0.15334000
H	-3.81407900	1.42978600	1.03888200
C	0.61120400	-1.00948900	2.90015600
H	0.34681400	-1.95914100	3.37205400
H	1.66867100	-0.81889300	3.10614300
H	0.00914000	-0.22609600	3.36818600

TS8-9

C	0.33138600	-1.26303300	0.99317800
C	-1.38407700	0.72920200	-0.29655800
C	-1.11133500	-1.09757000	1.51391500
C	-2.08973500	-0.30432900	0.61640100
H	-1.05052700	-0.59114400	2.48325300
H	-0.34393400	0.35404900	-0.49083500
H	-1.50739600	-2.09717700	1.72581900
H	-2.70134800	0.28827300	1.30858200
C	-0.11104300	2.25045400	1.16003200
H	0.01308900	3.17311200	1.71870200
C	0.99077300	0.00804100	0.57674100
C	0.56237100	-2.17048100	-0.22954800
H	0.46576600	-3.22993400	0.01870800
H	-0.17841700	-1.93822100	-0.99927300
C	1.95324100	-1.77057800	-0.72354000
H	2.13983600	-2.06038200	-1.75810900
H	2.73597800	-2.22741300	-0.11173500
C	1.96251800	-0.23171600	-0.55376600
H	1.48449100	0.19081500	-1.45421200
C	3.32643100	0.49726800	-0.43374600
C	4.39161500	-0.21376600	-1.27305500
H	4.08546900	-0.26545100	-2.32311100
H	5.32715700	0.34876500	-1.22793600
H	4.59642800	-1.22728700	-0.92307800
C	-3.12284900	-1.05147000	-0.26974400
H	-3.98504800	-1.29815100	0.35809100
C	-2.14983400	0.70377700	-1.63556900
H	-2.25628200	1.69283300	-2.08405800
H	-1.60365100	0.08665300	-2.35765300
C	-3.49342000	0.03819100	-1.29170000
H	-3.99967400	-0.36232300	-2.17350100
H	-4.16266500	0.77406400	-0.83274400
C	-2.68259200	-2.33945700	-0.96543100
H	-2.00912700	-2.15107800	-1.80709700
H	-2.19304400	-3.04082000	-0.28300500
H	-3.56128900	-2.84486700	-1.37342900
C	0.97886700	1.22049500	1.43597400
H	1.95360900	1.71142200	1.39227800
H	0.82463800	0.88275100	2.47021200
C	3.20434100	1.95313300	-0.89826800
H	3.08113500	1.97892900	-1.98644900
H	2.34864700	2.48157600	-0.46723900
H	4.10857900	2.51476500	-0.65362200
H	3.64647500	0.46567700	0.61725600
C	-1.17789600	2.05581800	0.38508500
C	-2.24743400	3.10469700	0.24120800
H	-2.06122200	3.95130600	0.90281600
H	-2.30113900	3.47934600	-0.78474600
H	-3.23024300	2.68721100	0.48643000
C	1.21645200	-1.77795400	2.18030100
H	0.90935100	-2.80693300	2.38287900
H	2.28475900	-1.78091400	1.95475800
H	1.05291700	-1.18378400	3.08058300

INT9

C	0.34076300	-1.33416500	0.84397900
C	-1.48593700	0.73048600	-0.37147100
C	-1.07596500	-1.17208000	1.42076600
C	-2.08566100	-0.28747100	0.64683900
H	-0.97202400	-0.77728600	2.43674600
H	-0.60288200	0.28862400	-0.84496600
H	-1.48553400	-2.18141700	1.54514600
H	-2.59060400	0.32216800	1.40671500
C	0.02410500	2.12169500	1.10339700
H	0.23078500	3.06821900	1.59474600
C	1.11357500	-0.10529600	0.64545000
C	0.54461900	-2.12071100	-0.46587000
H	0.43560100	-3.19714000	-0.31447900
H	-0.20534200	-1.81230900	-1.19876300
C	1.94496400	-1.70314300	-0.92933500
H	2.09527400	-1.82736600	-2.00189800
H	2.71971700	-2.28634100	-0.42381500
C	2.03893400	-0.21647900	-0.50730800
H	1.48792100	0.36728200	-1.27476300
C	3.42959000	0.45795000	-0.36791800
C	4.44184000	-0.18561100	-1.31909300
H	4.10730000	-0.09746500	-2.35783300
H	5.40027900	0.33181400	-1.23491800
H	4.61466200	-1.24105000	-1.10005900
C	-3.22938700	-1.01110700	-0.10056100
H	-3.95553700	-1.37559200	0.63441900
C	-2.57163100	0.86364800	-1.47901800
H	-2.78199000	1.89656500	-1.75520900
H	-2.22004100	0.36905100	-2.38854600
C	-3.81210000	0.13885000	-0.93289800
H	-4.48069400	-0.20432300	-1.72625200
H	-4.38622500	0.81130300	-0.28543800
C	-2.81807400	-2.18609200	-0.98658300
H	-2.21411900	-1.86518500	-1.84181500
H	-2.25609500	-2.94887800	-0.43826800
H	-3.70974200	-2.67131500	-1.39107700
C	1.01810300	1.04149700	1.55598100
H	2.00149700	1.50675000	1.66536500
H	0.68805800	0.69322500	2.54227800
C	3.34552300	1.96070500	-0.65622000
H	3.14624500	2.11945000	-1.72166800
H	2.55324300	2.47044100	-0.10116400
H	4.29323700	2.44902200	-0.41947800
H	3.77990700	0.29960600	0.66190300
C	-1.05244400	2.01841800	0.31699000
C	-1.93800200	3.22924400	0.16535400
H	-1.64899100	4.02196200	0.85622400
H	-1.89236400	3.63086300	-0.85111900
H	-2.98306200	2.96590500	0.36231200
C	1.24670800	-2.00122900	1.96363800
H	0.86887700	-3.02153900	2.06805700
H	2.30569300	-2.05598300	1.70349900
H	1.13981300	-1.48372000	2.91739700

TS9-10

C	0.32558100	-1.51170600	0.59202800
C	-1.44833400	0.73452900	-0.38383600
C	-1.11154600	-1.46260000	1.12900500
C	-2.02011800	-0.28582000	0.67245700
H	-1.07812400	-1.47215700	2.22288300
H	-0.64526300	0.27187600	-0.96757800
H	-1.55779900	-2.42135000	0.84870400
H	-2.22842800	0.31390100	1.56579800
C	0.16540100	1.95742500	1.13483500
H	0.52027400	2.88570400	1.57208700
C	1.09353200	-0.27298100	0.62226900
C	0.55353500	-2.08506700	-0.81891100
H	0.47693100	-3.17485700	-0.82619800
H	-0.21188800	-1.69897100	-1.50029500
C	1.94767000	-1.57099200	-1.20028200
H	2.09602600	-1.49039300	-2.27730900
H	2.72395800	-2.24019100	-0.81846900
C	2.05564200	-0.19148400	-0.50311900
H	1.55024100	0.55317300	-1.15323100
C	3.47108900	0.37512500	-0.20383700
C	4.45113300	-0.04058700	-1.30534000
H	4.11804300	0.33464900	-2.27868200
H	5.43304300	0.39235700	-1.10161500
H	4.57525700	-1.12251600	-1.37686600
C	-3.40404500	-0.72384100	0.12202200
H	-4.06730100	-0.97844200	0.95551300
C	-2.62942000	0.99392800	-1.35756100
H	-2.68425100	2.02277000	-1.71097600
H	-2.50653700	0.36902800	-2.24635500
C	-3.87479700	0.55085600	-0.58743500
H	-4.74102600	0.38673200	-1.23265600
H	-4.15082900	1.31013900	0.15443200
C	-3.35862000	-1.91292000	-0.84217200
H	-2.58671200	-1.79509300	-1.61095600
H	-3.17634200	-2.86091100	-0.33022300
H	-4.31824300	-2.01202800	-1.35583500
C	0.89939300	0.72451500	1.67975000
H	1.87652600	1.04996500	2.05135500
H	0.33088800	0.29434800	2.51118500
C	3.46945700	1.90228700	-0.09135500
H	3.19988200	2.34623100	-1.05579300
H	2.77209000	2.29229700	0.65238000
H	4.46811200	2.25840200	0.17095800
H	3.81233900	-0.06270200	0.74520300
C	-0.86323100	1.98508500	0.27851000
C	-1.48332300	3.32473100	-0.02587100
H	-1.04078200	4.11066700	0.58724300
H	-1.34607600	3.59568000	-1.07724500
H	-2.56109100	3.30727600	0.16511200
C	1.21820400	-2.33729200	1.61521000
H	0.77721500	-3.33788400	1.62258100
H	2.26350300	-2.42498400	1.31418900
H	1.16315900	-1.92185100	2.62246100

INT10

C	0.35114800	-1.61640600	0.25933700
C	-1.51659200	0.70953100	-0.51828400
C	-1.17931700	-1.69130800	0.46911200
C	-1.94330200	-0.36121900	0.54892400
H	-1.41010200	-2.28367400	1.36098300
H	-0.80690000	0.27988900	-1.24105200
H	-1.55345000	-2.27274900	-0.37943600
H	-1.77473200	0.08242800	1.53331900
C	0.15841700	1.81120900	1.03539700
H	0.61261500	2.71860700	1.42018700
C	1.08389700	-0.37951400	0.52752000
C	0.84599100	-2.06949100	-1.12748900
H	0.78068200	-3.15391200	-1.24325500
H	0.20944800	-1.61753600	-1.89676300
C	2.26924800	-1.52398600	-1.21439100
H	2.61234200	-1.37640400	-2.23840700
H	2.98039600	-2.19848800	-0.72707800
C	2.20060700	-0.18979700	-0.43557400
H	1.73182500	0.55545000	-1.11617000
C	3.54001700	0.40891100	0.06462700
C	4.64008600	0.15469800	-0.97268400
H	4.38786500	0.63227700	-1.92562700
H	5.57948100	0.58810500	-0.62324500
H	4.81372300	-0.90718500	-1.15497200
C	-3.49243200	-0.51775000	0.42959600
H	-3.91782900	-0.61731600	1.43336500
C	-2.82811800	1.04557400	-1.26219400
H	-2.84301500	2.04204900	-1.70334100
H	-2.95929500	0.33791300	-2.08583600
C	-3.91127600	0.81511300	-0.20996400
H	-4.92098500	0.78501400	-0.62653200
H	-3.88247400	1.61733400	0.53829600
C	-3.97325700	-1.71070500	-0.39983800
H	-3.56559000	-1.70753100	-1.41631000
H	-3.71196100	-2.66577700	0.06379800
H	-5.06224200	-1.68058600	-0.48370300
C	0.75562900	0.52803700	1.63436700
H	1.67662600	0.79887800	2.15945500
H	0.07456500	0.05431800	2.34472400
C	3.46790700	1.91362800	0.34164400
H	3.14765100	2.45108600	-0.55755300
H	2.79022400	2.18607400	1.15117100
H	4.45901200	2.28351800	0.61358900
H	3.81175400	-0.11922400	0.99032500
C	-0.82121500	1.91281800	0.12518100
C	-1.23724600	3.29588100	-0.30351500
H	-0.65278400	4.06117100	0.20856400
H	-1.10293300	3.42572900	-1.38247100
H	-2.29550400	3.47237800	-0.08834600
C	1.09687000	-2.47605300	1.37427000
H	0.81699000	-3.51085600	1.16033100
H	2.18718000	-2.40866700	1.34501400
H	0.74504200	-2.20733600	2.37072300

TS10-11

C	0.34988400	-1.55823100	0.15345500
C	-1.70549600	0.74313700	-0.51883500
C	-1.18002000	-1.66315600	0.35344800
C	-1.98643400	-0.37322400	0.53841700
H	-1.39810200	-2.33271800	1.19257800
H	-1.17718900	0.32037100	-1.38804100
H	-1.54973100	-2.17714700	-0.53980900
H	-1.78048700	0.05600300	1.52225300
C	0.19833700	1.76071700	0.87252500
H	0.80064900	2.64467400	1.05806900
C	1.10053500	-0.34945800	0.49726100
C	0.86209600	-1.94972300	-1.24387500
H	0.75917500	-3.02225100	-1.42537200
H	0.26437400	-1.42741600	-1.99972100
C	2.30701500	-1.45673700	-1.26069800
H	2.68657700	-1.27201700	-2.26579700
H	2.97453000	-2.18577600	-0.79091100
C	2.26741300	-0.15891700	-0.41926400
H	1.88607200	0.63899100	-1.08680500
C	3.61142600	0.33012900	0.17806500
C	4.74155500	0.08952000	-0.83006700
H	4.55362600	0.64270400	-1.75668900
H	5.68603500	0.44804500	-0.41552100
H	4.86942700	-0.96516300	-1.07933900
C	-3.52340900	-0.61225500	0.52943500
H	-3.85039000	-0.89258200	1.53649500
C	-3.11682600	1.19814600	-0.98828200
H	-3.18469200	2.25825000	-1.22682400
H	-3.37903900	0.65881400	-1.90213100
C	-4.05034900	0.77564700	0.14582600
H	-5.10280600	0.76502400	-0.14755900
H	-3.94548900	1.46447100	0.99294700
C	-4.00612900	-1.69027700	-0.44385100
H	-3.64030800	-1.52762100	-1.46340200
H	-3.69531200	-2.69134500	-0.13332600
H	-5.09815600	-1.68919800	-0.48246900
C	0.73074000	0.52156300	1.61333800
H	1.60697200	0.82011700	2.19118400
H	0.00338200	0.05729500	2.27770800
C	3.60568100	1.81536000	0.55518500
H	3.33756900	2.42783000	-0.31278000
H	2.92505000	2.06824900	1.36952300
H	4.60592200	2.11616700	0.87474400
H	3.81655700	-0.27119300	1.07577500
C	-0.81152100	1.87187300	-0.00979500
C	-1.03688300	3.23962300	-0.60618400
H	-0.22453100	3.92372200	-0.35848900
H	-1.12139700	3.17782900	-1.69599700
H	-1.96940700	3.67657200	-0.23679600
C	1.08327600	-2.46469700	1.24314900
H	0.82564300	-3.49081000	0.96723700
H	2.17275100	-2.38285000	1.25340500
H	0.69738100	-2.25128200	2.24036500

INT11

C	0.35322400	-1.44915500	-0.01891600
C	-2.10828500	0.87371800	-0.27137500
C	-1.12264000	-1.45735700	-0.44922300
C	-2.03914500	-0.52501800	0.33433600
H	-1.46471300	-2.49451300	-0.34941500
H	-2.54053100	0.76209000	-1.27864300
H	-1.19513600	-1.21691900	-1.51816000
H	-1.70107400	-0.45094600	1.37462400
C	0.40079200	1.41252400	-0.05656300
H	1.16886000	2.09757800	-0.39741100
C	1.07197600	-0.09450300	0.21711200
C	1.24068400	-2.10128500	-1.09386800
H	1.11585100	-3.18682200	-1.11753100
H	0.96353600	-1.71178100	-2.08122800
C	2.65371700	-1.65516400	-0.74322600
H	3.36182000	-1.79875600	-1.56084900
H	3.03419200	-2.21105200	0.12016900
C	2.49610700	-0.16047900	-0.39551400
H	2.46177500	0.37890600	-1.35372300
C	3.64903900	0.43401400	0.44180600
C	4.99820400	-0.08842600	-0.06727500
H	5.13879500	0.17366000	-1.12193300
H	5.81366300	0.36752400	0.49882900
H	5.09158800	-1.17118500	0.03038800
C	-3.54019300	-0.89134400	0.42201400
H	-3.67441800	-1.66336600	1.18579700
C	-3.19333200	1.58414800	0.58172200
H	-2.73923800	1.95511500	1.50653000
H	-3.64340400	2.43466400	0.06608800
C	-4.20405600	0.44154500	0.87011300
H	-5.13722400	0.60583000	0.32579100
H	-4.45297800	0.42302000	1.93292400
C	-4.12820300	-1.41682000	-0.88879900
H	-4.05103200	-0.68923800	-1.70425900
H	-3.64110400	-2.34027300	-1.21288000
H	-5.19070700	-1.63486200	-0.75619700
C	0.73783400	0.75492600	1.32850900
H	1.51036200	1.32854400	1.82971300
H	-0.11708400	0.51446800	1.94713500
C	3.69893700	1.96828900	0.41188000
H	3.79363600	2.32096800	-0.62168800
H	2.83278700	2.46510600	0.85712900
H	4.57572600	2.32438800	0.95771600
H	3.52105500	0.09398900	1.48017500
C	-0.88278800	1.66928100	-0.51181300
C	-1.07456000	2.90597200	-1.32864400
H	-0.16366300	3.21066800	-1.84563800
H	-1.89870000	2.80723700	-2.03699600
H	-1.34164200	3.71372800	-0.63191400
C	0.49294800	-2.22476000	1.30839100
H	0.24985700	-3.27752500	1.14181700
H	1.50568100	-2.17096700	1.71645100
H	-0.18810200	-1.83991200	2.07319700

TS11-12

C	0.37302100	-1.42744400	0.07588700
C	-2.07649300	0.85233200	-0.23948700
C	-1.10075000	-1.54964700	-0.33792400
C	-2.04913000	-0.56616500	0.34104600
H	-1.41281700	-2.57463400	-0.10065000
H	-2.42085900	0.77420500	-1.28285700
H	-1.18240000	-1.44639700	-1.42819700
H	-1.77825400	-0.47260000	1.40356000
C	0.42226700	1.05662900	-0.89786900
H	1.12711700	1.75000600	-1.35769800
C	1.02647600	-0.05927400	-0.21581900
C	1.28572600	-2.32539700	-0.78571800
H	1.15033300	-3.38273800	-0.54448500
H	1.03536600	-2.18794500	-1.84490100
C	2.69441700	-1.80804700	-0.50148000
H	3.42547400	-2.12988800	-1.24496500
H	3.04255500	-2.17280800	0.46839100
C	2.54241500	-0.27062500	-0.49152000
H	2.71927800	0.07958600	-1.51868800
C	3.52895900	0.49377400	0.41874300
C	4.93120300	-0.11985300	0.33558000
H	5.28960700	-0.11544600	-0.69962800
H	5.63330200	0.46776300	0.93173300
H	4.96354300	-1.14684100	0.70258100
C	-3.54989600	-0.93897100	0.35022800
H	-3.72185000	-1.69689800	1.12085500
C	-3.18854200	1.51685400	0.58152700
H	-2.79101500	1.80740100	1.56263700
H	-3.59166400	2.41687400	0.11260600
C	-4.25001400	0.39821200	0.72714400
H	-5.09517900	0.58192500	0.05856800
H	-4.64994100	0.37277000	1.74236500
C	-4.04934500	-1.49658600	-0.98295200
H	-3.89956400	-0.78999500	-1.80686600
H	-3.55251700	-2.43448800	-1.24614300
H	-5.12166200	-1.69813000	-0.92272300
C	0.45280500	1.17998600	0.71082300
H	1.10996200	1.94656200	1.09903100
H	-0.16819600	0.66238400	1.43152400
C	3.64128100	1.97495800	0.03400400
H	4.06246000	2.06517000	-0.97331500
H	2.69366900	2.52253200	0.03878500
H	4.31301300	2.49806000	0.71860100
H	3.17593000	0.40964700	1.45752500
C	-0.78071500	1.63163500	-0.38246700
C	-0.94691400	3.11990800	-0.58943000
H	0.00674700	3.61827400	-0.77675800
H	-1.60768400	3.29319600	-1.44363600
H	-1.41454700	3.57385200	0.28659900
C	0.56453700	-1.80340800	1.55627300
H	0.36614100	-2.87055500	1.68644800
H	1.58054300	-1.60529100	1.90500900
H	-0.11473300	-1.26877600	2.22515500

INT12

C	0.47140500	-1.38947000	0.19344600
C	-2.06395900	0.73839900	-0.37653700
C	-1.01458900	-1.58206500	-0.14576500
C	-2.01767200	-0.58177000	0.40381300
H	-1.26938700	-2.58886500	0.21074900
H	-2.11287700	0.49357400	-1.44528700
H	-1.11485800	-1.61320600	-1.23764100
H	-1.80366900	-0.37147300	1.46165800
C	0.60698900	1.21762200	-0.18509400
H	1.27348900	1.95811600	-0.60899900
C	1.12234800	-0.08021100	-0.15993300
C	1.31852700	-2.39211600	-0.63062400
H	1.24022100	-3.40128200	-0.21839900
H	0.93097800	-2.42317900	-1.65470600
C	2.73784300	-1.83515300	-0.61877000
H	3.32021000	-2.15588200	-1.48334600
H	3.27843300	-2.16591400	0.27418500
C	2.55685900	-0.30349400	-0.57415300
H	2.64294900	0.13431100	-1.58309800
C	3.65801000	0.38006900	0.30516500
C	5.02218300	0.04641000	-0.30927100
H	5.11024500	0.49309600	-1.30553800
H	5.82021600	0.45712800	0.31324100
H	5.19197300	-1.02794800	-0.39971800
C	-3.48929000	-1.05416700	0.36185300
H	-3.65594700	-1.80356300	1.14317400
C	-3.40892300	1.38981100	0.05514700
H	-3.23069700	2.17797400	0.79452800
H	-3.90309500	1.85992600	-0.79820800
C	-4.25258000	0.24568600	0.67072900
H	-5.27014500	0.22464000	0.27407800
H	-4.33167600	0.38394800	1.75247100
C	-3.90782900	-1.65470800	-0.98168200
H	-3.79726300	-0.93988700	-1.80434100
H	-3.33367200	-2.55138500	-1.23075100
H	-4.96131100	-1.94183000	-0.94404100
C	-0.20844500	1.80215700	1.04863800
H	0.22415800	2.74516300	1.37013900
H	-0.41725200	1.09127700	1.83788100
C	3.54384700	1.89264000	0.48814900
H	3.51743100	2.41199600	-0.47680300
H	2.67291800	2.18829400	1.07667000
H	4.42631500	2.25255900	1.02248000
H	3.59972500	-0.09147600	1.29550000
C	-0.92042500	1.71572900	-0.20540500
C	-1.03561700	2.96513800	-1.05817000
H	-0.19121500	3.64234600	-0.91077500
H	-1.09958000	2.70284400	-2.11684500
H	-1.94758900	3.50122400	-0.78300400
C	0.74400200	-1.61488100	1.70659200
H	0.44081200	-2.63716400	1.94892100
H	1.80168100	-1.50691800	1.95939900
H	0.17234800	-0.93220500	2.33765400

1,3-shift-Forward

C	1.32283400	2.15697100	-0.10381700
C	2.11512900	0.99980000	0.07345100
C	-1.08710100	0.08364800	0.20646300
C	1.95870500	-0.37984800	-0.48152300
C	-0.41723700	-1.31300800	0.12760200
C	1.07718500	-1.25744000	0.47439800
H	3.89559400	-0.32448600	-1.39260100
H	1.47748500	-2.27460500	0.43006000
H	1.19574500	-0.92169700	1.51238900
C	0.07018400	2.13095100	-0.68688200
H	-0.39000800	3.11256600	-0.81325500
C	-0.78310500	0.97456900	-1.04476100
H	-1.69198700	1.33904600	-1.51855300
H	-0.27243600	0.36966900	-1.80232700
C	-1.21028700	-2.07531400	1.20294100
H	-1.03493100	-3.15430100	1.17082800
H	-0.91838100	-1.71730900	2.19897200
C	-2.65747900	-1.67970900	0.90896600
H	-3.33369500	-1.89956000	1.73757700
H	-3.02504000	-2.23605200	0.04078200
C	-2.59380800	-0.16502500	0.60664600
H	-2.74777700	0.36044900	1.55874400
C	3.43635200	-0.86816800	-0.55534600
H	1.50325300	-0.36553800	-1.47381700
C	4.03321000	-0.31504600	0.74229500
H	3.72319500	-0.92877500	1.59495100
H	5.12363800	-0.28478800	0.73321200
C	3.40748500	1.07946900	0.81971200
H	3.26575000	1.49885100	1.82367900
H	4.02708200	1.82221500	0.28924100
C	3.64467500	-2.35760200	-0.79341000
H	3.03865500	-2.71807400	-1.63012900
H	4.69230000	-2.54669600	-1.03780900
H	3.40567300	-2.95506800	0.08970700
C	1.86889200	3.48313000	0.38630000
H	2.83011200	3.71938400	-0.07858200
H	1.17890700	4.29527000	0.15625000
H	2.01761400	3.47293400	1.46979000
C	-3.68468900	0.36116300	-0.34138200
H	-3.41388500	0.10173200	-1.37651100
C	-3.83274100	1.88314100	-0.20947900
H	-4.47843800	2.28957000	-0.99213500
H	-4.29156500	2.12042300	0.75614200
H	-2.87966100	2.42139300	-0.24420800
C	-5.04440600	-0.27916300	-0.04307200
H	-5.05207700	-1.35413700	-0.23312900
H	-5.32058300	-0.11675800	1.00464700
H	-5.82090000	0.17429400	-0.66455800
H	-0.62982600	0.58206600	1.06863500
C	-0.62763300	-2.00782200	-1.22885800
H	0.06077100	-1.66922300	-2.00865500
H	-0.47074900	-3.08514300	-1.12031800
H	-1.64082100	-1.85583300	-1.61047400

1,3-shift-TS

C	1.47051000	1.96136000	-0.23707500
C	2.04479300	0.68399600	0.26678800
C	-0.99956900	-0.07354700	0.03782100
C	1.84311200	-0.68150500	-0.40680500
C	-0.53944900	-1.55029500	-0.18546300
C	0.93595300	-1.73757800	0.20897300
H	3.23459100	0.77862000	-0.14599500
H	1.28148200	-2.72716100	-0.11572200
H	1.01890200	-1.71756700	1.30058900
C	0.27066300	2.00269500	-0.84266800
H	-0.07239700	2.99885700	-1.12051100
C	-0.67507100	0.87233700	-1.15391100
H	-1.58698500	1.31066300	-1.55722400
H	-0.25970600	0.30190000	-1.99051200
C	-1.47540500	-2.33115100	0.75316800
H	-1.46651400	-3.40745200	0.55833100
H	-1.16478700	-2.17749000	1.79552900
C	-2.83057900	-1.66806300	0.52815200
H	-3.56339400	-1.92950900	1.29444600
H	-3.25095400	-1.97509300	-0.43675900
C	-2.50115100	-0.16332900	0.53013000
H	-2.49516500	0.15414200	1.58263600
C	3.30933600	-0.64901300	-0.10858500
H	1.67715800	-0.55492300	-1.47832500
C	3.50857100	-0.67622800	1.40122100
H	3.29124900	-1.67069600	1.80025900
H	4.52192900	-0.39631200	1.69272600
C	2.45838900	0.41304600	1.72736100
H	1.61045300	0.09178100	2.33458500
H	2.90823600	1.28900900	2.19576500
C	4.36455000	-0.92178400	-1.12927700
H	4.07693300	-0.57015900	-2.12169200
H	5.33478100	-0.51454100	-0.84346500
H	4.44926700	-2.01580400	-1.17751000
C	2.27324300	3.20429100	0.05509400
H	3.26862400	3.15554900	-0.40196700
H	1.77037600	4.08810100	-0.33863900
H	2.41221600	3.35345900	1.13056700
C	-3.54189000	0.72519200	-0.16498000
H	-3.45609700	0.58950000	-1.25336400
C	-3.32844300	2.20069100	0.19251500
H	-3.96966100	2.85126800	-0.40799800
H	-3.58562300	2.36143000	1.24511000
H	-2.29306300	2.53137000	0.06285700
C	-4.96469000	0.32168200	0.23442000
H	-5.21964300	-0.68554100	-0.10250600
H	-5.08095400	0.35595500	1.32361300
H	-5.69214800	1.01301300	-0.19903300
H	-0.43036300	0.30119000	0.89889500
C	-0.75915800	-2.04027400	-1.62718600
H	0.01740700	-1.70180100	-2.32060700
H	-0.75090900	-3.13385000	-1.65129100
H	-1.71753400	-1.70304900	-2.03095500

1,3-shift-Reverse

C	1.77751900	2.13330900	-0.07908000
C	2.55177000	0.88869000	-0.45807500
C	-1.11698300	0.46397800	0.46152100
C	1.80821200	-0.43891500	-0.68208500
C	-0.53465900	-0.96554500	0.41507200
C	0.99433800	-0.96247500	0.63314300
H	3.06655700	1.10559000	-1.40761100
H	1.28179200	-1.97825900	0.94731700
H	1.27464800	-0.29085400	1.44756700
C	0.46016900	2.33110300	-0.20131900
H	0.11021200	3.31164700	0.11817500
C	-0.64859100	1.41272700	-0.66340900
H	-1.47905000	2.04417000	-0.98378000
H	-0.36567600	0.85322100	-1.56068000
C	-1.22702100	-1.57959000	1.64603800
H	-1.15574200	-2.67193500	1.66433500
H	-0.75455600	-1.19992300	2.55957100
C	-2.68100400	-1.07347900	1.53231600
H	-3.11212500	-0.90650400	2.52103100
H	-3.30166100	-1.83128300	1.05327000
C	-2.64078900	0.24883000	0.70034700
H	-2.98871400	1.06116600	1.34726400
C	2.76981200	-1.48356100	-0.50481800
H	1.16436800	-0.53158900	-1.55203400
C	3.95286900	-0.97959500	0.23575000
H	4.25119600	-1.62433900	1.06849400
H	4.76887800	-1.05859700	-0.50434200
C	3.64268100	0.48673700	0.57014500
H	3.26255900	0.57338400	1.59181100
H	4.52840300	1.11823600	0.50124800
C	2.67882600	-2.84598100	-1.06032200
H	1.67622500	-3.09282900	-1.40819000
H	3.34856200	-2.84688500	-1.93394000
H	3.06161700	-3.60275600	-0.37180600
C	2.66889500	3.25429000	0.40312600
H	3.46985900	3.45489000	-0.31830800
H	2.10095400	4.17542200	0.53673700
H	3.14935300	3.01453700	1.35774100
C	-3.51824400	0.32259600	-0.58806600
H	-2.86926200	0.18213400	-1.46422500
C	-4.15807600	1.71333000	-0.69524400
H	-4.65877800	1.84573200	-1.65777500
H	-4.90837200	1.83659700	0.09355200
H	-3.43181400	2.52253700	-0.57709400
C	-4.63285600	-0.72437400	-0.66965500
H	-4.26028000	-1.74519900	-0.79496800
H	-5.25832100	-0.70229800	0.22911900
H	-5.27750700	-0.51106000	-1.52634200
H	-0.73470500	0.88767200	1.40041800
C	-0.94142800	-1.77069900	-0.82318200
H	-0.67304700	-1.29844600	-1.77309000
H	-0.49447800	-2.77104900	-0.79206200
H	-2.02372700	-1.91138900	-0.84463100

4. Experiment Details

General Information

All reagents and solvents were purchased from either Aldrich Chemical Company, Inc., Kanto Kagaku Co., Inc., Merck & Co., Inc., Nacalai Tesque Company, Ltd., or Tokyo Kasei Kogyo Co., Ltd., and used without further purification unless otherwise indicated. Dichloromethane (CH_2Cl_2) was distilled from phosphoric pentaoxide (P_2O_5). Tetrahydrofuran (THF), *N*,*N*-dimethylformamide (DMF), and diethyl ether (Et_2O) of anhydrous grade were used. All reactions were performed under argon. FTIR spectra were measured on a JASCO FT/IR-6200 infrared spectrophotometer. ^{1}H NMR spectra were recorded on an either Bruker AVANCE 300 (300 MHz) or spectrometer. Chemical shifts of ^{1}H NMR were reported in parts per million (ppm, d) relative to $CHCl_3$ (d = 7.26) in $CDCl_3$. ^{13}C NMR spectra were recorded on an either Bruker AVANCE 300 (75 MHz), JEOL JNM-LA 400 (100 MHz), spectrometer. Chemical shifts of ^{13}C NMR were reported in ppm (d) relative to $CHCl_3$ (d = 77.0) in $CDCl_3$. Low resolution mass spectra (LRMS) and high resolution mass spectra (HRMS) were obtained on an JEOL JMS-AX500 using either fast atom bombardment ionization (FAB) or electroionizaion (EI) method. All reactions were monitored by thin layer chromatography (TLC), which was performed with precoated plates (silica gel 60 F-254, 0.25 mm thickness, manufactured by Merck). TLC visualization was accompanied using UV lamp (254 nm) or a charring solution (ethanoic phosphomolybdic acid). Daiso IR-60 1002W (40/63 mm) was used for flash column chromatography on silica gel.

(2*E*)-Ethyl-4-[[*t*-buthyldiphenylsilyl]oxy]-3-*d*-2-methylbuta-2-enoate (SI-3)

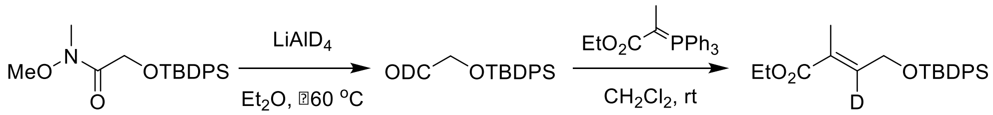

SI-1 **SI-2** **SI-3**

To a suspension of $LiAlD_4$ (587 mg, 14.0 mmol) in Et_2O (140 mL) was slowly added a solution of **SI-1**[8] (5.00 g, 14.0 mmol) in Et_2O at -60 °C. The mixture was stirred for 30 min at -60 °C, quenched with 1*N* NaOH (100 mL), filtered, and extracted with Et_2O (x3). The combined organic layers were washed with brine, dried over anhydrous $MgSO_4$, and filtered. The filtrate was concentrated under reduced pressure to give the crude **SI-2**, which was subjected to the next reaction without further purification.

To a solution of **SI-2** (4.19 g) in CH_2Cl_2 (140 mL) was added ethyl 2-(triphenylphosphoranylidene)propionate (6.16 g, 17 mmol) at room temperature. After stirred for 1 h, the mixture was concentrated under reduced pressure. The residue was purified by flash column chromatography on silica gel to give **SI-3** (3.38 g, 63%, 2 steps from **SI-1**).

FTIR (neat) 3072, 2958, 2933, 2858, 1713, 1428, 1302, 1279, 1200, 1166, 1113, 1064 cm^{-1}.

^{1}H NMR (300 MHz, $CDCl_3$) d 7.74-7.65 (m, 4 H), 7.49-7.36 (m, 6 H), 4.37 (s, 2 H), 4.21 (q, *J* = 7.1 Hz, 2 H), 1.66 (s, 3 H) 1.32 (t, *J* = 7.1 Hz, 3 H), 1.08 (s, 9 H), ^{13}C NMR (75 MHz, $CDCl_3$) d 167.6, 140.3 (t, *J* = 24.5 Hz), 135.5, 133.2, 129.7, 127.7, 127.4, 61.2, 60.5, 26.7, 19.1, 14.2, 12.5.

(2*E*)-4-[[*t*-Buthyldiphenylsilyl]oxy]-3-*d*-2-methylbuta-2-en-1-ol (SI-4)

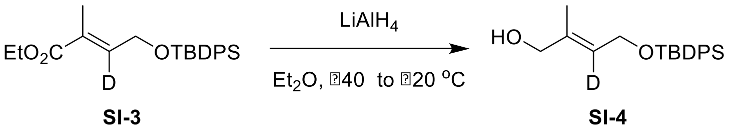

To a suspension of $LiAlH_4$ (592 mg, 15.6 mmol) in Et_2O (150 mL) was slowly added a solution of **SI-3** (3.99 g, 10.4 mmol) in Et_2O at -40 °C. The mixture was stirred for 1 h at -20 °C, quenched with H_2O (100 mL), acidified with 1*N* HCl (100 mL), and extracted with Et_2O (x3). The combined organic layers were washed with brine, dried over anhydrous $MgSO_4$, and filtered. The filtrate was concentrated under reduced pressure. The residue was purified by flash column chromatography on silica gel to give **SI-4** (2.91 g, 82%).

FTIR (neat) 3371, 2931, 2858, 1473, 1428, 1112, 1075, 1008 cm^{-1}

^{1}H NMR (300 MHz, $CDCl_3$) d 7.84-7.65 (m, 4 H), 7.49-7.36 (m, 6 H), 4.30 (s, 2 H), 3.97 (s, 2 H), 1.50 (s, 3 H), 1.07 (s, 9 H), ^{13}C NMR (75 MHz, $CDCl_3$) d 136.5, 135.9, 134.2, 130.0, 128.0, 124.7 (t, *J* = 23.7 Hz), 68.3, 61.1, 27.2, 19.5, 14.1.

(2*E*,6*E*,10*E*)-2-*d*-3,7,11,15-Tetramethyl-5-(phenylsulfonyl)hexadeca-2,6,10,14-tetraen-1-ol (SI-7)

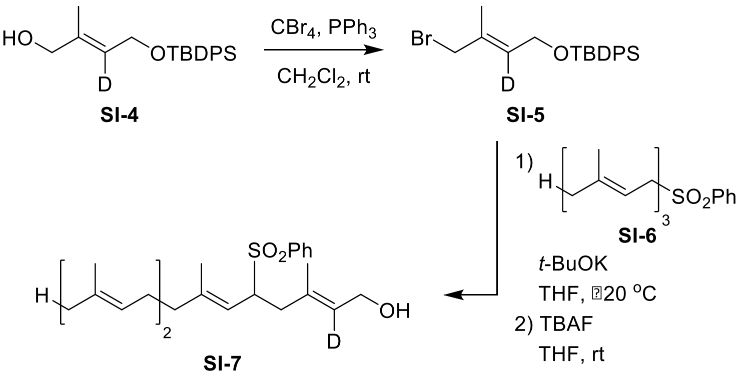

To a solution of **SI-4** (202 mg, 0.59 mmol) in CH_2Cl_2 (6 mL) was added CBr_4 (255 mg, 0.77 mmol) and PPh_3 (202 mg, 0.77 mmol) at 0 °C. The mixture was stirred at room temperature for 30 min and filtered to remove insoluble materials. The filtrate was concentrated under reduced pressure to give the crude **SI-5**, which was subjected to the next reaction without further purification.

To a suspension of *t*-BuOK (269 mg, 2.4 mmol) in THF (3 mL) was added a solution of **SI-6**[9] (308 mg, 0.89 mmol) in THF (1.5 mL) at -20 °C. The mixture was stirred for 15 min. A solution of **SI-5** (239 mg) in THF (1.5 mL) was added. The mixture was stirred for 1 h, quenched with aq. NH_4Cl, and extracted with AcOEt (x3). The combined organic layers were washed with brine, dried over anhydrous $MgSO_4$, and filtered. The filtrate was concentrated under reduced pressure to give the crude product, which was subjected to the next reaction without further purification.

To a solution of the crude product (395 mg) in THF (6 mL) was added TBAF (2.4 mmol, 1.0 M THF solution) at 0 °C. After stirred for 3 h at room temperature, the mixture was concentrated under reduced pressure. The residue was purified by flash column chromatography on silica gel to give **SI-7** (148 mg, 58%, 3 steps from **SI-4**).

FTIR (neat) 3439, 2966, 2918, 1659, 1447, 1382, 1304, 1146, 1085 cm^{-1}

^1H NMR (300 MHz, $CDCl_3$) d 7.84 (d, J = 7.3 Hz, 2 H), 7.63 (t, J = 7.3 Hz, 1 H), 7.51 (t, J = 7.3 Hz, 2 H), 5.14-4.98 (m, 2 H), 4.93 (d, J = 10.4 Hz 1 H), 4.09 (d, J = 10.8 Hz, 2 H), 3.91 (td, J = 10.8, 3.1 Hz, 1 H), 2.93 (dd, J = 13.6, 3.0 Hz, 1 H), 2.34 (dd, J = 13.6, 11.3 Hz, 1 H), 2.11-1.87 (m, 8 H), 1.68 (s, 3 H), 1.61 (s, 3 H), 1.58 (s, 3 H), 1.48 (s, 3 H), 1.17 (s, 3 H); ^{13}C NMR (75 MHz, $CDCl_3$) d 144.8, 138.5, 137.7, 133.2, 131.6, 129.8, 129.0, 128.5, 127.3 (t, J = 22.8 Hz), 123.5, 123.4, 117.0, 63.0, 58.7, 39.5, 37.0, 26.5, 26.1, 25.5, 17.5, 16.2, 16.0, 15.8.

(2*E*,6*E*,10*E*)-2-*d*-3,7,11,15-Tetramethylhexadeca-2,6,10,14-tetraen-1-ol (SI-8)

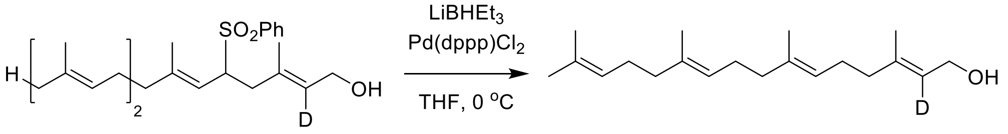

SI-7 **SI-8**

To a solution of **SI-7** (1.81 g, 4.2 mmol) and $Pd(dppp)Cl_2$ (124 mg, 0.21 mmol) in THF (85 mL) was added $LiBHEt_3$ (21 mmol, 1.0 M THF solution) at 0 °C. The mixture was stirred for 1 h at 0 °C, quenched with aq. NH_4Cl, and extracted with AcOEt (x3). The combined organic layers were washed with brine, dried over anhydrous $MgSO_4$, and filtered. The filtrate was concentrated under reduced pressure. The residue was purified by flash column chromatography on silica gel to give **SI-8** (1.14 g, 93%).

FTIR (neat) 3342, 2966, 2925, 2857, 1653, 1438, 1376, 1107, 1006 cm^{-1}

^1H NMR (300 MHz, $CDCl_3$) d 5.16-5.05 (m, 3 H), 4.15 (s, 2 H), 2.17-1.92 (m, 12 H), 1.68 (s, 6 H), 1.60 (s, 9 H); ^{13}C NMR (75 MHz, $CDCl_3$) d 139.1, 135.2, 134.8, 131.0, 124.3, 124.1, 123.7 123.0 (t, J = 23.4 Hz), 59.0, 39.59, 39.56, 39.4, 26.6, 26.5, 26.2, 25.6, 25.5, 17.5, 16.1, 15.8

(4*E*)-6-[[*t*-Buthyldiphenylsilyl]oxy]-1-*d*-4-methylhexa-4-enal (SI-10)

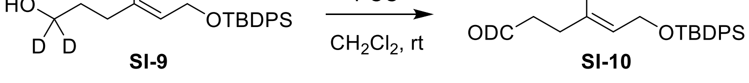

SI-9 **SI-10**

To a solution of **SI-9**[10] (7.08 g, 19.1 mmol) in CH_2Cl_2 (190 mL) was added PCC (8.23 g, 38.2 mmol) at room temperature. After stirred for 3 h, the suspension was filtered through a celite pad. The filtrate was concentrated under reduced pressure. The residue was purified by flash column chromatography on silica gel to give **SI-10** (5.05 g, 72%).

FTIR (neat) 3072, 2931, 2858, 2072, 1715, 1472, 1428, 1112, 1063 cm^{-1}

^1H NMR (300 MHz, $CDCl_3$) d 7.70-7.65 (m, 4 H), 7.46-7.34 (m, 6 H), 5.38 (t, J = 6.2 Hz, 1 H), 4.21 (d, J = 6.2 Hz, 2 H), 2.49 (t, J = 7.5 Hz, 2 H), 2.29 (t, J = 7.5 Hz, 2 H), 1.44 (s, 3 H), 1.04 (s, 9 H), ^{13}C NMR (75 MHz, $CDCl_3$) d 201.6 (t, J = 25.9 Hz), 135.5, 134.9, 133.8, 129.5, 127.5, 124.9, 60.9, 41.5 (t, J = 3.6 Hz), 31.3, 26.8, 19.1, 16.3

(2*E*,6*E*)-Ethyl-8-[[*t*-buthyldiphenylsilyl]oxy]-3-*d*-2,6-dimethylocta-2,6-dienoate (SI-11)

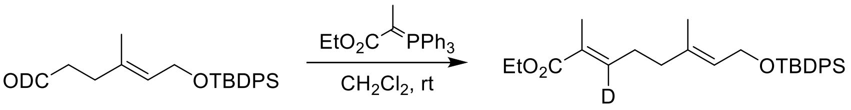

SI-10 **SI-11**

To a solution of **SI-10** (5.04 g, 13.7 mmol) in CH_2Cl_2 (137 mL) was added ethyl 2-(triphenylphosphoranylidene)propionate (7.43 g, 20.5 mmol) at room temperature. After stirred for 2 h, the mixture was concentrated under reduced pressure. The residue was purified by flash column chromatography on silica gel to give **SI-11** (5.14 g, 83%).

FTIR (neat) 2932, 2858, 1710, 1638, 1428, 1230, 1277, 1196, 1064, 1112, 1064 cm^{-1}

^1H NMR (300 MHz, $CDCl_3$) d 7.71-7.63 (m, 4 H), 7.45-7.34 (m, 6 H), 5.39 (t, *J* = 6.2 Hz, 1 H), 4.25-4.13 (m, 4 H), 2.25 (t, *J* = 7.6 Hz, 2 H), 2.08 (t, *J* = 7.6 Hz, 2 H), 1.83 (s, 3 H), 1.45 (s, 3 H), 1.28 (t, *J* = 7.2 Hz 3 H), 1.04 (s, 9 H); ^{13}C NMR (75 MHz, $CDCl_3$) d 168.2, 141.3 (t, *J* = 24.0 Hz), 136.0, 135.7, 134.1, 129.6, 127.7, 124.8, 61.2, 60.5, 38.0, 27.0, 26.9, 19.2, 16.4, 14.4, 12.5.

(2*E*,6*E*)-8-[[*t*-Buthyldiphenylsilyl]oxy]-3-*d*-2,6-dimethylocta-2,6-dien-1-ol (SI-12)

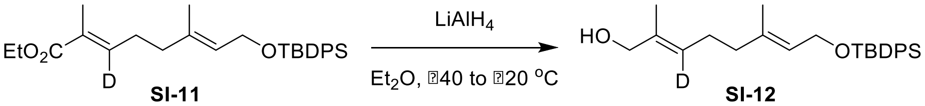

SI-11 **SI-12**

To a suspension of $LiAlH_4$ (861 mg, 22.7 mmol) in Et_2O (114 mL) was slowly added a solution of **SI-11** (5.15 g, 11.4 mmol) in Et_2O at -40 °C. The mixture was stirred for 1 h at -20 °C, quenched with H_2O (100 mL), acidified with 1*N* HCl (100 mL), and extracted with Et_2O (x3). The combined organic layers were washed with brine, dried over anhydrous $MgSO_4$, and filtered. The filtrate was concentrated under reduced pressure. The residue was purified by flash column chromatography on silica gel to give **SI-12** (3.27 g, 70%).

FTIR (neat) 3387, 2931, 2858, 1428, 1112, 1066, cm^{-1}

^1H NMR (300 MHz, $CDCl_3$) d 7.72-7.65 (m, 4 H), 7.46-7.34(m, 6 H), 5.38 (t, *J* = 6.2 Hz, 1 H), 4.22 (d, *J* = 6.4 Hz, 2 H), 3.99 (s, 2 H), 2.12 (t, *J* = 7.6 Hz 2 H), 2.01 (t, *J* = 7.6 Hz 2 H), 1.67 (s, 3 H), 1.45 (s, 3 H), 1.04 (s, 9 H); ^{13}C NMR (75 MHz, $CDCl_3$) d 136.8, 135.6, 134.9, 134.1, 129.6, 127.7, 125.3 (t, *J* = 23.1 Hz), 124.3, 68.8, 61.2, 39.1, 26.9, 25.8, 19.2, 16.4, 13.7.

(2*E*,6*E*,10*E*)-6-*d*-3,7,11,15-Tetramethyl-9-(phenylsulfonyl)hexadeca-2,6,10,14-tetraen-1-ol (SI-15)

To a solution of **SI-12** (3.27 g, 7.99 mmol) in CH_2Cl_2 (80 mL) was added CBr_4 (3.45 g, 10.4 mmol) and PPh_3 (2.73 g, 10.4 mmol) at 0 °C. The mixture was stirred at room temperature for 30 min and filtered to remove insoluble materials. The filtrate was concentrated under reduced pressure to give the crude **SI-13**, which was subjected to the next reaction without further purification.

To a suspension of *t*-BuOK (3.59 g, 32. 0mmol) in THF (40 mL) was added a solution of **SI-14** (3.34 g, 12.0 mmol) in THF (20 mL) at -20 °C. The mixture was stirred for 15 min. A solution of **SI-13** (3.78 g) in THF (20 mL) was added. The mixture was stirred for 1 h, quenched with aq. NH_4Cl, and extracted with AcOEt (x3). The combined organic

layers were washed with brine, dried over anhydrous $MgSO_4$, and filtered. The filtrate was concentrated under reduced pressure to give the crude product, which was subjected to the next reaction without further purification.

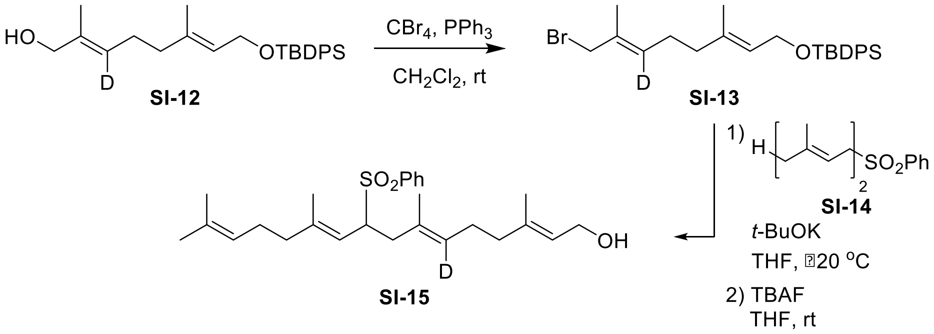

To a solution of the crude product (5.35 g) in THF (80 mL) was added TBAF (32.0 mmol, 1.0 M THF solution) at 0 °C. After stirred for 2 h at room temperature, the mixture was concentrated under reduced pressure. The residue was purified by flash column chromatography on silica gel to give **SI-15** (2.17 g, 63%, 3 steps from **SI-12**).

FTIR (neat) 3437, 2970, 2919, 2856, 1447, 1304. 1145. 1085 cm^{-1}

^{1}H NMR (300 MHz, $CDCl_3$) d 7.84 (d, *J* = 7.3 Hz, 2 H), 7.62 (t, *J* = 7.3 Hz, 1 H), 7.51 (t, *J* = 7.3 Hz, 2 H), 5.37 (t, *J* = 6.7 Hz, 1 H), 5.02 (m 1 H), 4.90 (d, *J* = 10.8 Hz 1 H), 4.13 (d, *J* = 6.7 Hz, 2 H), 3.88 (td, *J* = 11.0, 2.9 Hz, 1 H), 2.88 (dd, *J* = 13.2, 2.9 Hz, 1 H), 2.28 (dd, *J* = 13.2, 11.0 Hz, 1 H), 2.11-1.88 (m, 8 H), 1.68 (s, 3 H), 1.64 (s, 3 H), 1.58 (s, 3 H), 1.53 (s, 3 H), 1.17 (s, 3 H); ^{13}C NMR (75 MHz, $CDCl_3$) d 144.8, 138.5, 137.7, 133.2, 131.6, 129.8, 129.0, 128.5, 127.3 (t, *J* = 22.8 Hz), 123.5, 123.4, 117.0, 63.3, 58.9, 39.4, 38.9, 37.1, 26.04, 26.01, 25.4, 17.4, 16.1, 16.0, 15.7

(2*E*,6*E*,10*E*)-6-*d*-3,7,11,15-Tetramethylhexadeca-2,6,10,14-tetraen-1-ol (SI-16)

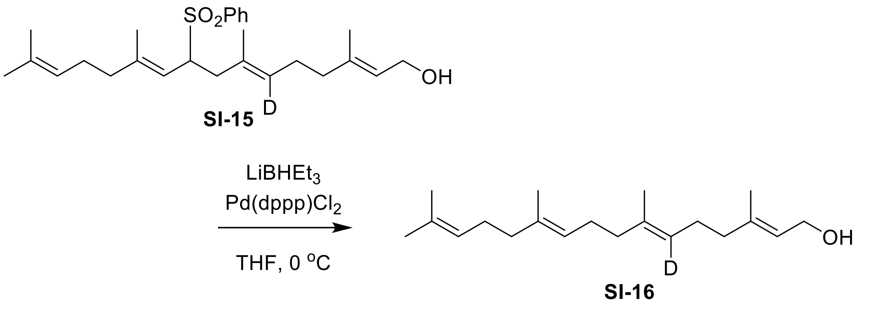

To a solution of **SI-15** (203 mg, 0.47 mmol) and Pd(dppp)Cl_2 (14 mg, 24 mmol) in THF (5 mL) was added $LiBHEt_3$ (2.4 mmol, 1.0 M THF solution) at 0 °C. The mixture was stirred for 1 h at 0 °C, quenched with aq. NH_4Cl, and extracted with AcOEt (x3). The combined organic layers were washed with brine, dried over anhydrous $MgSO_4$, and filtered. The filtrate was concentrated under reduced pressure. The residue was purified by flash column chromatography on silica gel to give **SI-16** (103 mg, 75%).

FTIR (neat) 3343, 2966, 2919, 2856, 1669, 1447, 1376, 1105, 1001 cm^{-1}

^{1}H NMR (300 MHz, $CDCl_3$) d 5.42 (t, *J* = 7.0 Hz, 1 H), 5.15-5.06 (m, 2 H), 4.15 (d, *J* = 7.0 Hz, 2 H), 2.16-1.93 (m, 12 H), 1.68 (s, 6 H), 1.60 (s, 9 H); ^{13}C NMR (75 MHz, $CDCl_3$) d 139.7, 135.2, 134.9, 131.1, 124.3, 123.80 (t, *J* = 25.7 Hz), 123.76, 123.3, 59.2, 39.64, 39.60, 39.5, 26.7, 26.5, 26.4, 26.2, 26.0, 17.6, 16.2, 15.9

^{1}H-NMR data of (a) GGOH, (b) 2D-GGOH, and (c) 6D-GGOH

(a) GGOH

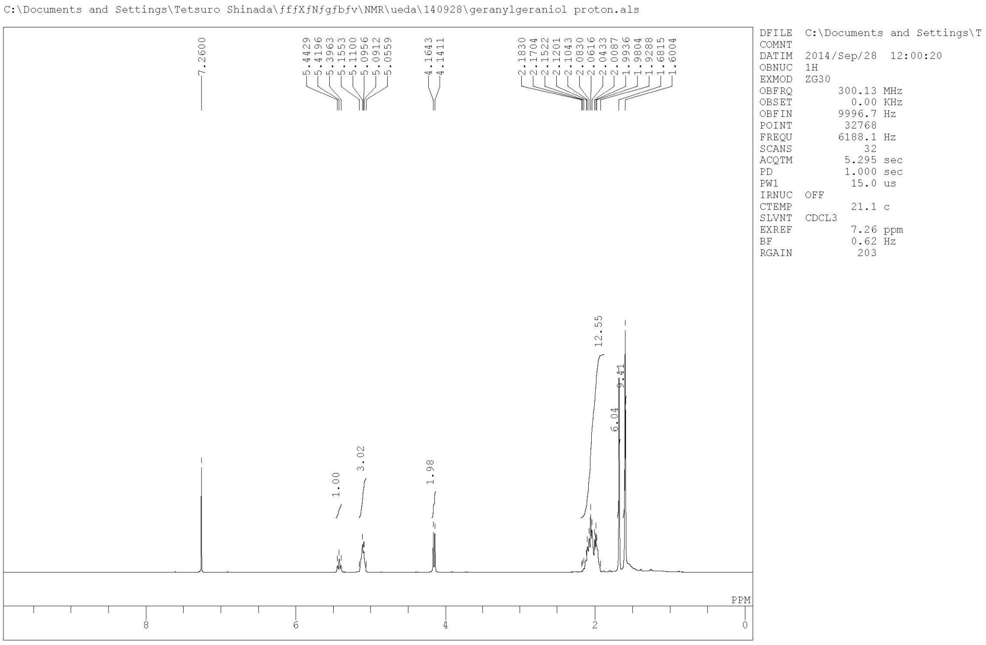

(b) 2D-GGOH

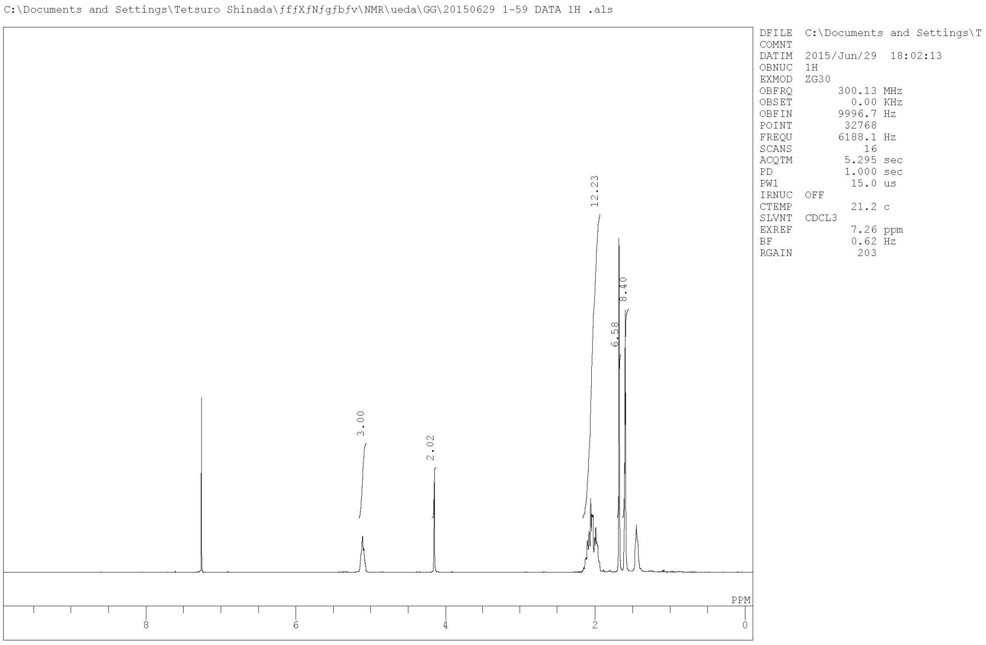

(c) 6D-GGOH

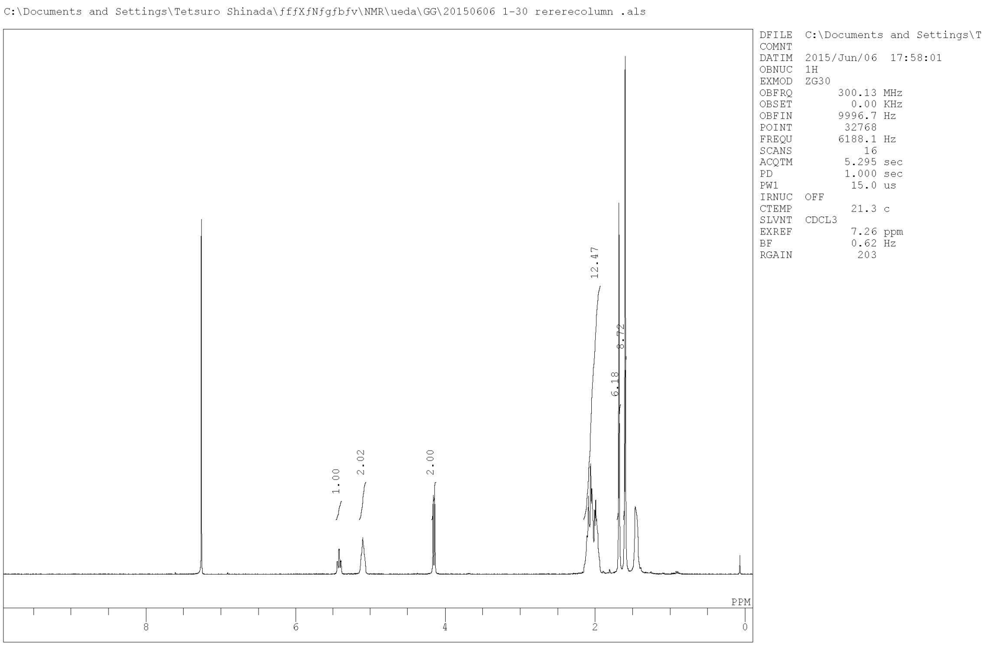

Deuterium Tracing Experiments

(a) Comparison of (3-D)-PD by ^{1}H NMR spectra with natural abundance PD.[11]

natural abundance PD

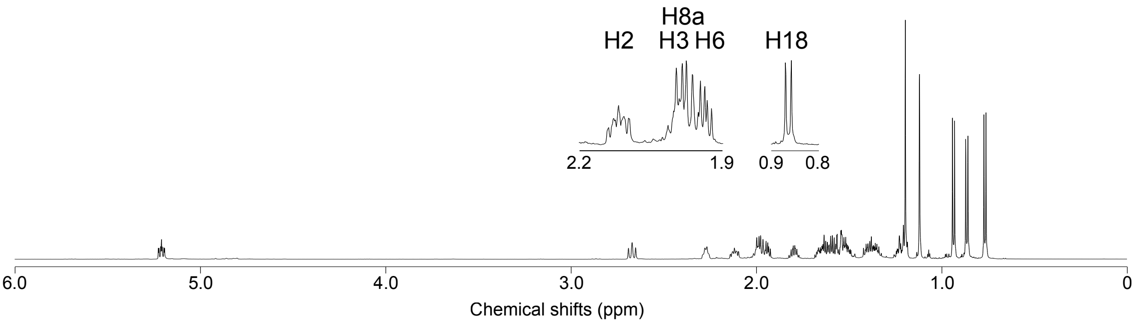

(3-D)-PD

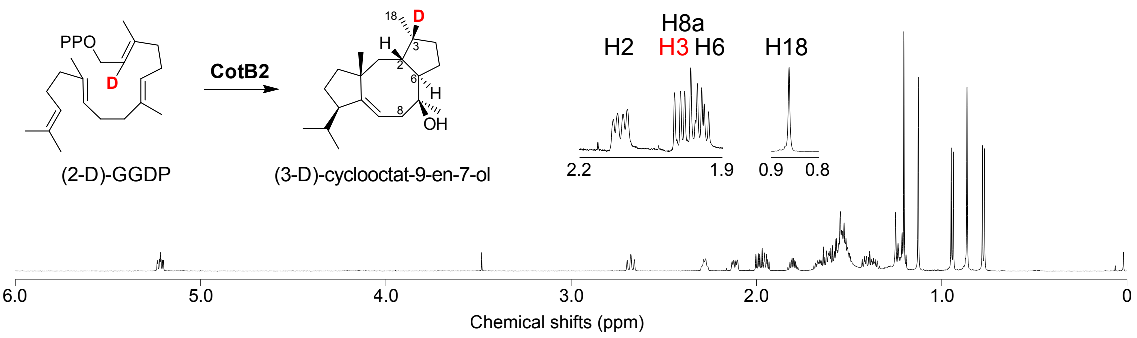

(b) Comparison of (2-D)-PD by ^{1}H NMR spectra with natural abundance PD.[11]

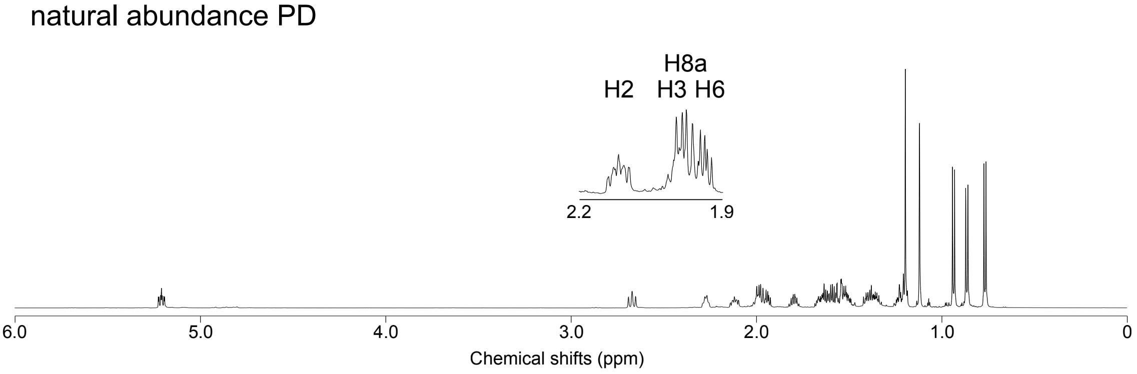

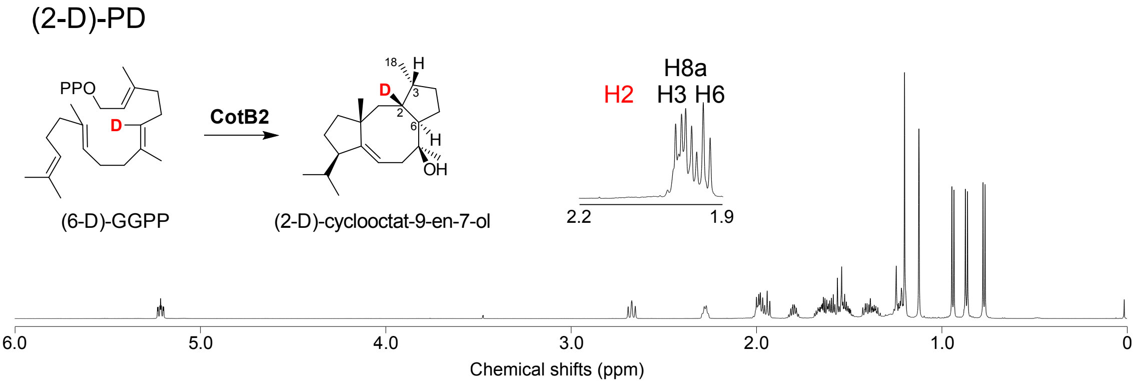

5. Reference

1. M. J. Frisch, G. W. Trucks, H. B. Schlegel, G. E. Scuseria, M. A. Robb, J. R. Cheeseman, G. Scalmani, V. Barone, B. Mennucci, G. A. Petersson, H. Nakatsuji, M. Caricato, X. Li, H. P. Hratchian, A. F. Izmaylov, J. Bloino, G. Zheng, J. L. Sonnenberg, M. Hada, M. Ehara, K. Toyota, R. Fukuda, J. Hasegawa, M. Ishida, T. Nakajima, Y. Honda, O. Kitao, H. Nakai, T. Vreven, J. A. Montgomery, Jr., J. E. Peralta, F. Ogliaro, M. Bearpark, J. J. Heyd, E. Brothers, K. N. Kudin, V. N. Staroverov, T. Keith, R. Kobayashi, J. Normand, K. Raghavachari, A. Rendell, J. C. Burant, S. S. Iyengar, J. Tomasi, M. Cossi, N. Rega, J. M. Millam, M. Klene, J. E. Knox, J. B. Cross, V. Bakken, C. Adamo, J. Jaramillo, R. Gomperts, R. E. Stratmann, O. Yazyev, A. J. Austin, R. Cammi, C. Pomelli, J. W. Ochterski, R. L. Martin, K. Morokuma, V. G. Zakrzewski, G. A. Voth, P. Salvador, J. J. Dannenberg, S. Dapprich, A. D. Daniels, O. Farkas, J. B. Foresman, J. V. Ortiz, J. Cioslowski, and D. J. Fox, *Gaussian 09, Revision D.01*, Gaussian, Inc., Wallingford CT, **2013**.
2. Y. Zhao, D. G. Truhlar, *Theor. Chem. Acc.* **2008**, *120*, 215.
3. (a) W. J. Hehre, R. Ditchfield, J. A. Pople, *J. Chem. Phys.* **1972**, *56*, 2257. (b) M. M. Francl, W. J. Petro, W. J. Hehre, J. S. Binkley, M. S. Gordon, D. J. DeFrees, J. A. Pople, *J. Chem. Phys.* **1982**, *77*, 3654.
4. (a) S. Maeda, Y. Osada, K. Morokuma, K. Ohno, *GRRM 11, Version 11.03*, **2012**. (b) S. Maeda, K. Ohno, K. Morokuma, *Phys. Chem. Chem. Phys.* **2013**, *15*, 3683. (c) K. Ohno, S. Maeda, *Chem. Phys. Lett.*, **2004**, *384*, 277. (d) S. Maeda, K. Ohno, *J. Phys. Chem. A*, **2005**, *109*, 5742. (e) K. Ohno, S. Maeda, *J. Phys. Chem. A*, **2006**, *110*, 8933.
5. The molecular geometries for the transition states were first estimated by Reaction plus software package (Reaction plus, HPC Systems Inc., http://www.hpc.co.jp/chem/ (written in Japanese)), based on the nudged elastic band (NEB)[6] and string[7] methods, and were re-optimized by Gaussian 09 subsequently.
6. H. Jonsson, G. Mills, K. W. Jacobsen, "Nudged elastic band method for finding minimum energy paths of transitions", in "Classical and Quantum Dynamics in Condensed Phase Simulations", ed. by B. J. Berne, G. Ciccotti, D. F. Coker, p.385 (World Scientific, Singapore, 1998)
7. E. Weinan, W. Ren, E. Vanden-Eijnden, *Phys. Rev. B.*, **2002**, *66*, 052301.
8. H. Nemoto, M. Nagamochi, H. Ishibashi, K. Hukumoto, *J. Org. Chem.* **1994**, *59*, 74.
9. (a) S. Bouzbouz, B. Kirschleger, *Synlett* **1994**, 763. (b) C. Tsangarakis, M. Stratakis, *Eur. J. Org. Chem.* **2006**, 4435.
10. C. A. Citron, P. Rabe, L. Barra, C. Nakano, T. Hoshino, J. S. Dickschat, *Eur. J. Org. Chem.* **2014**, 7684.
11. A. Meguro, Y. Motoyoshi, K. Teramoto, S. Ueda, Y. Totsuka, Y. Ando, T. Tomita, S-Y. Kim, T. Kimura, M. Igarashi, R. Sawa, T. Shinada, M. Nishiyama, T. Kuzuyama. *Angew. Chem. Int. Ed.* **2015**, *54*, 4353.
